# Anti-Ulcerative Colitis Effects and Active Ingredients in Ethyl Acetate Extract from Decoction of *Sargentodoxa cuneata*

**DOI:** 10.3390/molecules28227663

**Published:** 2023-11-19

**Authors:** Piao Yu, Feng Xu, Hongmei Wu, Xiangpei Wang, Qin Ding, Mei Zhang, Rongze Fang, Ping Qin

**Affiliations:** 1 Department of Pharmacy, Guizhou University of Traditional Chinese Medicine, Guiyang 550025, China; ypleen@126.com (P.Y.); whm0425@126.com (H.W.); a_012456789@sina.com (Q.D.); 18798793674@163.com (M.Z.); 18300851163@163.com (R.F.); qp08071045@163.com (P.Q.); 2 School of Chinese Ethnic Medicine, Guizhou Minzu University, Guiyang 550025, China; wxp0123@126.com

**Keywords:** *Sargentodoxa cuneata*, ulcerative colitis, gas chromatography and mass spectrometry, molecular docking, liquid chromatography–mass spectrometry, TLR4/NF-κB/NLRP3 pathway

## Abstract

Ulcerative colitis (UC) is an intractable disease prevalent worldwide. While ethyl acetate extract from decoction of *Sargentodoxa cuneata* (EAdSc) has potential anti-inflammatory activity, its effects on UC remain unknown. In this study, the constituent compounds discussed in the literature and identified by gas chromatography and mass spectrometry (GC–MS) were collected, and the blood-soluble components of EAdSc were identified by liquid chromatography–mass spectrometry. The network pharmacology analysis and molecular docking analysis were performed to explore the potential underlying mechanism and active ingredients of EAdSc against UC. Furthermore, mice with dextran sulfate sodium (DSS)-induced UC were used to study the therapeutic effects and validate the mechanism of EAdSc against UC. A total of 53 compounds from EAdSc were identified in the literature and by GC–MS, and 22 blood-soluble EAdSc components were recognized. Network pharmacology analysis revealed that multiple inflammatory signaling pathways are involved in EAdSc’s anti-UC activity. Furthermore, molecular docking analysis showed that the eleutheroside A, liriodendrin, epicatechin, 2-methoxy-4-vinylphenol, catechin, androsin, coumaroyltyramine, and catechol may be active against UC through the TLR4/NF-κB/NLRP3 pathway. EAdSc reduced the disease activity, macroscopic colon damage, and histological damage indices, as well as inhibiting DSS-induced spleen enlargement and colon shortening. In addition, EAdSc decreased the levels of tumor necrosis factor-α (TNF-α), interleukin (IL)-1β, IL-6, and IL-17, as well as the expression of TLR4, NF-κB p65, NLRP3, and Caspase-1 mRNA in colon tissues. These results provide insights into the anti-UC effects and underlying mechanisms of EAdSc and help elucidate the active ingredients of EAdSc in the treatment of UC.

## 1. Introduction

Ulcerative colitis (UC) is a chronic intestinal immune inflammatory disease, the course of which is protracted. As lifestyles continue to change, the worldwide prevalence of UC has increased annually [1]. Currently, the treatment drug options for UC primarily include amino salicylic acid, glucocorticoids, and certain biological agents. In the course of these treatments, various adverse reactions can occur, including drug dependence, cytotoxicity, and liver and kidney damage [2]. Additionally, relapse can easily occur after stopping the drug [3,4]. Therefore, it is important to identify novel, safe, and more effective drugs for the prevention and treatment of UC. Recent studies have shown that traditional Chinese medicines may provide a good alternative [5].

*Sargentodoxa cuneata* grows in sunny forests and is mainly distributed in the subtropical zone of China [6]. The dried cane of *Sargentodoxa cuneata* (Oliv.) has been reported to have many traditional uses in China, such as “clearing heat”, “detoxification”, and “pain relief” [7]. Additionally, it is often used to treat “intestinal carbuncles”, abdominal pain, and “heat toxin sores” [7]. Furthermore, *Sargentodoxa cuneata* is commonly used in the clinical treatment of UC [8,9,10,11]. Modern research has shown that *Sargentodoxa cuneata* shows significant anti-inflammatory activity and that its components, iriodendrin and 3,4-dihydroxyphenylethyl alcohol glycosides, reportedly show anti-UC properties [12,13]. In addition, ethyl acetate extract of *Sargentodoxa cuneata* has been confirmed to contain the effective anti-inflammatory components of the *Sargentodoxa cuneata* plant itself [14]. A total of 30 compounds have been isolated from ethyl acetate extract of *Sargentodoxa cuneata* [15,16]. While we propose that the ethyl acetate extract from decoction of *Sargentodoxa cuneata* (EAdSc) can be effectively used in the treatment of UC, this has not yet been confirmed.

In this study, the chemical constituents of EAdSc reported in the literature were collected, and the volatile components contained within the EAdSc were identified by gas chromatography and mass spectrometry (GC–MS), and the blood-soluble components of EAdSc were identified using liquid chromatography–mass spectrometry (LC–MS). Then, network pharmacology analysis and molecular docking analysis were performed. Further, mice with dextran sulfate sodium (DSS)-induced UC were used to study the therapeutic effects and validate the mechanism of EAdSc against UC. This study shows that EAdSc contains the active ingredients of *Sargentodoxa cuneata* effective in treating UC, the underlying mechanisms of which are related to the regulation of multiple inflammatory signaling pathways. This study also identified the anti-UC active ingredients in EAdSc that are conducive to the further development and utilization of *Sargentodoxa cuneata* in this context.

## 2. Results

### 2.1. Components of EAdSc Reported in the Literature and Those Identified by GC–MS

A total of 30 components of ethyl acetate extract of *Sargentodoxa cuneata* have been previously identified in the literature. In addition, another 29 chemical compounds were identified in EAdSc by GC–MS analysis (Table 1, Figure S1), and the identified compounds accounted for 53.96% of the total components. There were nine ingredients with contents higher than 2.0%. All components with druggability reported in the literature and those identified by GC–MS were included in the subsequent molecular docking analysis (Table 2).

### 2.2. Identification of Blood-Soluble EAdSc Components

After identification, it was found that there were 40 components with a total score higher than 80 in the control plasma and 57 components with a total score higher than 80 in the drug-containing plasma (Tables S1 and S2). By comparison, we retained only the components present in the drug-containing plasma that were not present in the control plasma. A total of 22 blood-soluble EAdSc components were obtained (Table 3). All blood-soluble EAdSc components were included in the network pharmacological analyses. The MS spectra are shown in Supplementary Figure S2. 

### 2.3. Network Analysis Results

**Figure 1:**
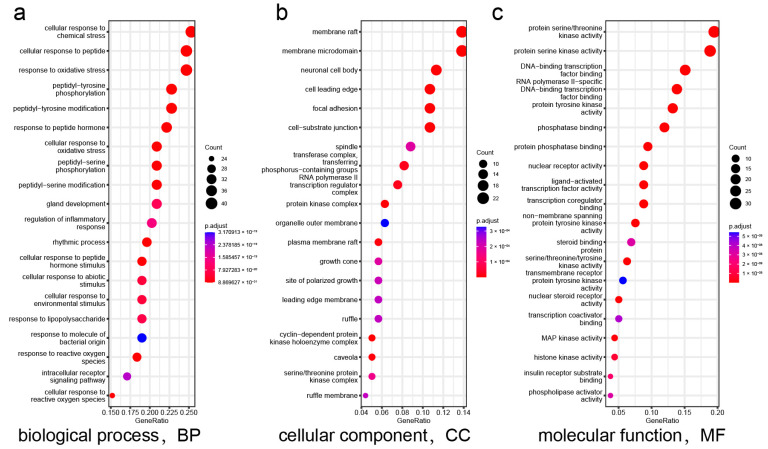
Results of the Gene Ontology (GO) enrichment analysis. The (**a**) biological processes, (**b**) cellular components, and (**c**) molecular functions identified in the GO enrichment analysis of intersection targets. The analyses are based on the core targets of EAdSc components and UC.

**Figure 2:**
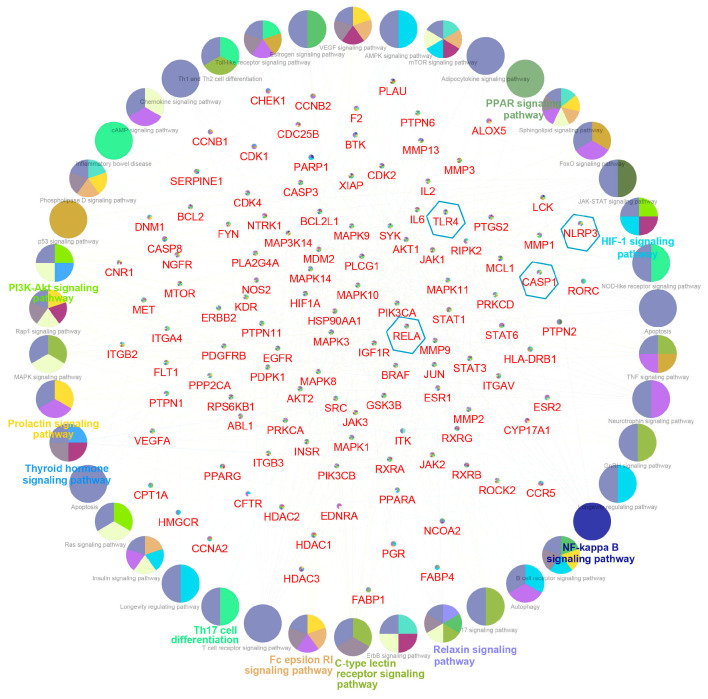
Results of the Kyoto Encyclopedia of Genes and Genomes (KEGG) pathway analysis. The core targets of the EAdSc component and UC are labeled in red font in the figure, while the targets within the blue hexagonal boxes are the core targets selected for validation in subsequent animal experiments in this study. The signaling pathways obtained by enrichment are presented as circles.

**Figure 3:**
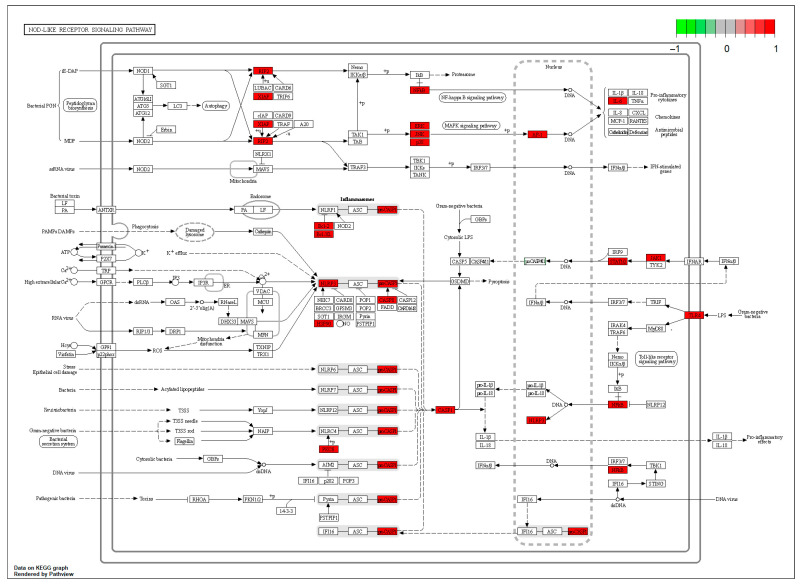
The direct targets of the ethyl acetate extract from decoction of *Sargentodoxa cuneata* (EAdSc) action on the NOD-like receptor signaling pathway. The targets are marked in red.

The “component–target–disease” interaction network of EAdSc was constructed, and 524 common targets of components and diseases were identified (Figure S3). Further, the screened core targets included MAPK1, MAPK3, SRC, HSP90AA1, STAT3, AKT, PIK3CA, ESR1, EGFR, MAPK14, and RELA (Table S3). GO analysis revealed that the EAdSc components primarily regulate biological processes such as cellular response to chemical stress, cellular response to peptide, response to oxidative stress, and regulation of inflammatory response (Figure 1a). EAdSc components also regulate membrane composition and adhesion (Figure 1b), as well as influencing protease activity and DNA and RNA transcription (Figure 1c). Moreover, the main inflammatory signaling pathways involved were the PI3K-Akt signaling pathway, IL-17 signaling pathway, JAK-STAT signaling pathway, NF-kappa B signaling pathway, TNF signaling pathway, and NOD-like receptor signaling pathway (Figure 2). The direct targets of EAdSc action on the NOD-like receptor signaling pathway are shown in Figure 3.

### 2.4. EAdSc Compounds Potentially Effective through the TLR4/NF-κB/NLRP3 Pathway

**Figure 4:**
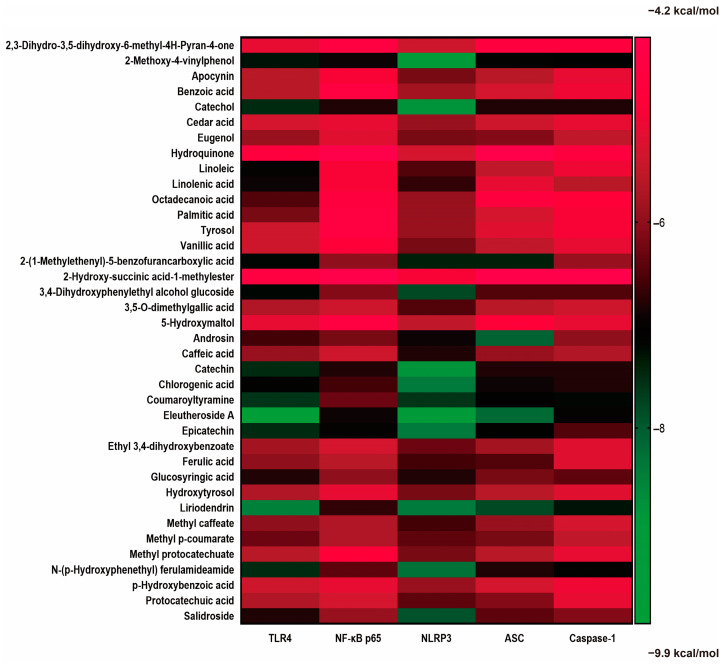
Results of molecular docking analysis of small-molecule compounds found in the ethyl acetate extract from decoction of *Sargentodoxa cuneata* (EAdSc) with the key proteins (TLR4, NF-κB-p65, NLRP3, ASC, and Caspase-1) of the TLR4/NF-κB/NLRP3 signaling pathway. The darker green color in the heatmap means that the compound has a strong affinity for the target, and the darker red color means that the compound has a weak affinity for the target.

The affinities of the potential compounds from EAdSc identified in the literature and by GC–MS for various core targets (TLR4, NF-κB-p65, NLRP3, ASC, and Caspase-1) were analyzed using molecular docking (Figure 4). According to the results, there were many components of EAdSc, such as eleutheroside A, liriodendrin, epicatechin, 2-methoxy-4-vinylphenol, catechin, androsin, coumaroyltyramine, and catechol, that showed good affinity for the core targets of the TLR4/NF-κB/NLRP3 pathway (Figure S4). These results suggest that these components have the potential to bind directly to the proteins TLR4, NF-κB-p65, NLRP3, ASC, and Caspase-1, which in turn inhibit TLR4/NF-κB/NLRP3 pathway activation, reduce the release of inflammatory factors, and alleviate UC. This also validates to some extent the results of the network pharmacology analysis.

### 2.5. EAdSc Ameliorated DSS-Induced UC Symptoms in Mice

**Figure 5:**
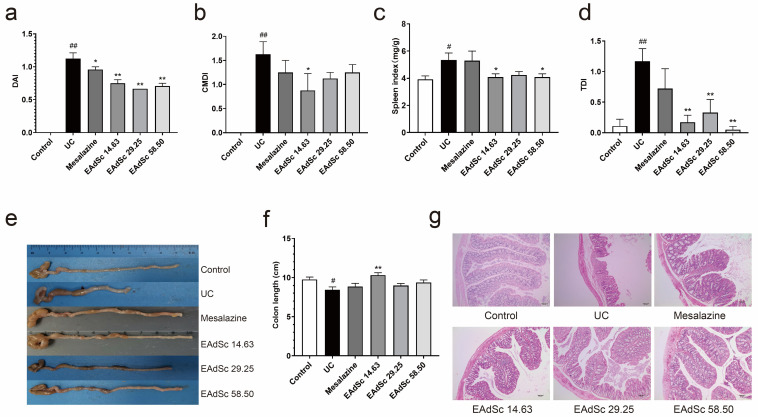
Results showing the anti-ulcerative colitis effects of the ethyl acetate extract from decoction of *Sargentodoxa cuneata* (EAdSc), including the (**a**) disease activity index (DAI) on the 10th day, (**b**) colon macroscopic damage index (CMDI), (**c**) spleen index, (**d**) histological damage index (TDI), (**e**) colon condition of the animals in each group, (**f**) colon length of the animals in each group, and (**g**) HE-stained pathological sections of the colons of the animals in each group. The mesalazine group was administered 0.52 g/kg mesalazine by gavage, and the EAdSc groups were dosed by intragastric gavage with 14.63 mg/kg, 29.25 mg/kg, and 58.50 mg/kg EAdSc. Data are presented as means ± SEM. n = 8 except TDI index n = 6, ^#^ *p* < 0.05 vs. the control group, ^##^ *p* < 0.01 vs. the control group, * *p* < 0.05 vs. the UC group, ** *p* < 0.01 vs. the UC group. SEM: standard error of mean.

Compared to those in the control group, animals in the UC group showed typical clinical symptoms of UC, such as weight loss, diarrhea, and bloody stools. After treatment with mesalazine (0.52 g/kg) or EAdSc (14.63 mg/kg, 29.25 mg/kg, 58.50 mg/kg), the DAI scores of mice with UC were significantly reduced on Day 10 (*p* < 0.05) (Figure 5a). In addition, treatment with 14.63 mg/kg EAdSc improved the changes in colon length and CMDI scores induced by DSS exposure. Although both mesalazine (0.52 g/kg) and EAdSc (29.25 mg/kg, 58.50 mg/kg) may have inhibited colon shortening and reduced CMDI scores, there were no statistically significant differences observed (Figure 5b,e,f). Furthermore, treatment with EAdSc (14.63 mg/kg, 58.50 mg/kg) significantly inhibited DSS-induced enlargement of the spleen (*p* < 0.05) (Figure 5c).

### 2.6. EAdSc Attenuated Histopathological Injury in Mice with DSS-Induced UC

Pathological changes, such as necrosis and shedding of epithelial cells, lamina propria cell necrosis, muscle fiber necrosis, inflammatory cell infiltration, and fibrous tissue proliferation, were all observed in the colon tissue of the mice within the UC group, indicating that a model of UC was successfully established (Figure 5g). Compared to those of the UC group, the TDI scores of the mice within the EAdSc groups were significantly lower (*p* < 0.01) (Figure 5d). Indeed, treatment with EAdSc significantly alleviated DSS-induced pathological damage (Figure 5g). EAdSc ameliorates the inflammatory response of UC colonic tissues and maintains the integrity of intestinal mucosal tissues.

### 2.7. EAdSc Inhibited Inflammation in Colon Tissues of Mice with DSS-Induced UC

**Figure 6:**
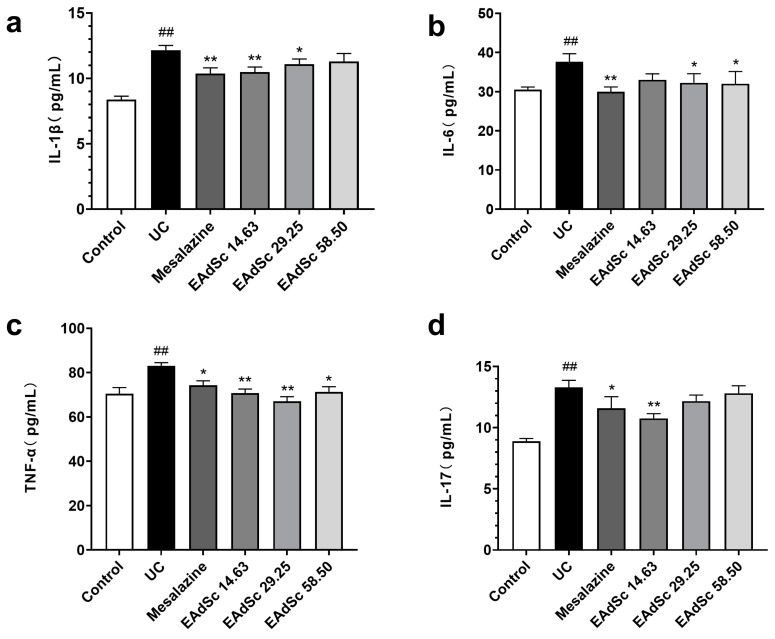
Ethyl acetate extract from decoction of *Sargentodoxa cuneata* (EAdSc) decreased the levels of inflammatory factors, including (**a**) IL-1β, (**b**) IL-6, (**c**) TNF-α, and (**d**) IL-17, in the colon tissues of animals with ulcerative colitis using ELISA analysis. The mesalazine group was administered 0.52 g/kg mesalazine by gavage, and the EAdSc groups were dosed by intragastric gavage with 14.63 mg/kg, 29.25 mg/kg, and 58.50 mg/kg EAdSc. Data are presented as means ± SEM. n = 8, ^##^ *p* < 0.01 vs. the control group, * *p* < 0.05 vs. the UC group, ** *p* < 0.01 vs. the UC group. SEM: standard error of the mean.

Levels of IL-1β, IL-6, TNF-α, and IL-17 in the colon tissues of the UC group were remarkably elevated compared to those of the control group (*p* < 0.01) (Figure 6). EAdSc (58.50 mg/kg, 29.25 mg/kg, and 14.63 mg/kg) treatment significantly reduced the expression of TNF-α in the colon tissues of UC animals (*p* < 0.05) (Figure 6c). In addition, 58.50 mg/kg and 29.25 mg/kg treatments of EAdSc significantly decreased the expression of IL-6 in the colon tissues of the animals (*p* < 0.05) (Figure 6b). Finally, treatment with 14.63 mg/kg of EAdSc significantly downregulated the expression of IL-1β and IL-17 in the colon tissues (Figure 6a,d). EAdSc has a good inhibitory effect on UC intestinal inflammation, and regulating intestinal immunoinflammation when EAdSc is used to treat UC is an important measure.

### 2.8. EAdSc Decreased the Expression of TLR4, NF-κB p65, NLRP3, and Caspase-1 mRNA in Colon Tissues of Mice with UC

**Figure 7:**
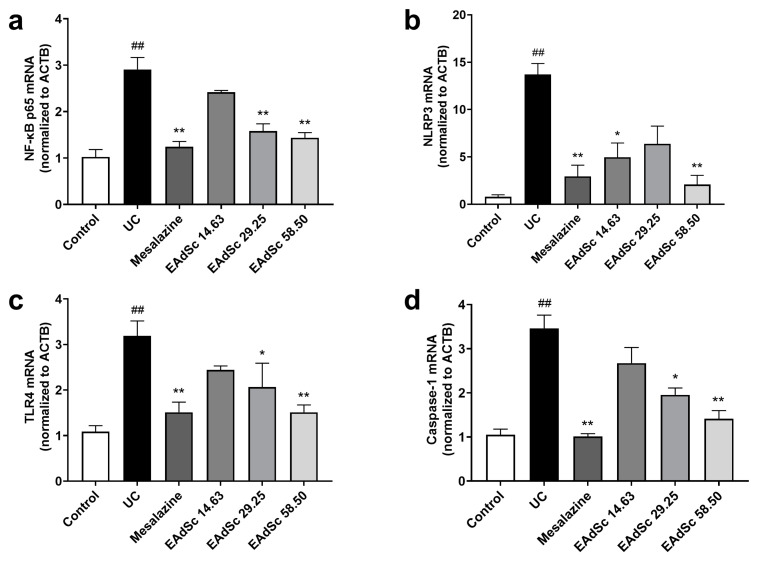
Effects of the ethyl acetate extract from decoction of *Sargentodoxa cuneata* (EAdSc) on (**a**) NF-κB p65, (**b**) NLRP3, (**c**) TLR4, and (**d**) Caspase-1 mRNA expressions in colon tissues. The mesalazine group was administered 0.52 g/kg mesalazine by gavage, and the EAdSc groups were dosed by intragastric gavage with 14.63 mg/kg, 29.25 mg/kg, and 58.50 mg/kg EAdSc. Data are presented as means ± SEM. n = 5, ^##^ *p* < 0.01 vs. the control group, * *p* < 0.05 vs. the UC group, ** *p* < 0.01 vs. the UC group. SEM: standard error of mean.

Compared to those of the control group, EAdSc (58.50 mg/kg) treatment decreased the expression of TLR4, NF-κB p65, NLRP3, and Caspase-1 mRNA in colon tissues (*p* < 0.05) (Figure 7). However, treatment with 29.25 mg/kg of EAdSc only decreased the expression of TLR4, NF-κB p65, and Caspase-1 mRNA in the colon tissues (*p* < 0.05) (Figure 7a,c,d). Finally, treatment with 14.63 mg/kg of EAdSc only reduced NLRP3 mRNA expression (*p* < 0.05) (Figure 7b). EAdSc significantly down-regulated gene expression of key targets in the TLR4/NF-κB/NLRP3 signaling pathway in UC colon tissues, suggesting that EAdSc inhibited the activation of the TLR4/NF-κB/NLRP3 signaling pathway. In addition, the results of animal experiments suggest that the “component–target–disease” network constructed in this study is highly reliable and can be used as a reference for subsequent studies.

## 3. Discussion

*Sargentodoxa cuneata* has been reported to have richer pharmacological activities, such as anti-inflammatory, antioxidant, anti-UC, anti-tumor, and anti-rheumatoid arthritis effects [6,17]. There are also reports of studies utilizing network pharmacology to explore the mechanism of action of *Sargentodoxa cuneata* ‘s anti-inflammatory and anti-UC effects [18,19]. However, the reports on the pharmacological activity studies of the ethyl acetate extract of *Sargentodoxa cuneata* were only seen in the studies of antioxidant and anti-inflammatory activities [14], and there were relatively few reports on the pharmacological studies of the ethyl acetate extract of *Sargentodoxa cuneata*, with the main reports focusing on the chemical studies of the ethyl acetate extract of *Sargentodoxa cuneata*. This suggests the need to strengthen the research on the mechanism and material basis of the anti-UC action of *Sargentodoxa cuneata*. EAdSc is an important part extracted from the decoction of *Sargentodoxa cuneata*. In this study, the non-blood-soluble and blood-soluble components of EAdSc were identified by using GC–MS and LC–MS, and combined with the literature reports, we explored the molecular mechanism and material basis of EAdSc anti-UC activity using network pharmacology and molecular docking. Further, animal experiments were applied for validation studies. These results were favorable for the elucidation of the mechanism of EAdSc’s anti-UC action and the material basis of its pharmacodynamic effects.

According to the serum pharmacochemistry method of traditional Chinese medicine, the components entering the blood and their metabolites may be the basis for this potent substance ’s efficacy [20]. To better understand the pharmacodynamics of substances in EAdSc for the treatment of UC, we used the serum pharmacochemistry of traditional Chinese medicine to conduct the study. A total of 22 blood-soluble EAdSc components were identified by LC–MS. Surprisingly, no blood-soluble EAdSc components have been previously reported in *Sargentodoxa cuneata*. A comparison with the current literature revealed that the bioavailability of most of the ingredients reported in EAdSc is poor. Therefore, we speculate that the ingredients in EAdSc may exert their effects on UC in two ways. First, some of the components of EAdSc are absorbed into the blood, thus improving UC through the bloodstream. Second, EAdSc may act directly on the epithelial cells of the colon or affect the intestinal flora. However, further studies are required to confirm this hypothesis.

Network pharmacology can be used to explore the therapeutic effects and mechanisms of action of drugs. To explore the molecular mechanisms of EAdSc compounds against UC, we performed network pharmacology analysis of compounds from EAdSc identified in the literature and by GC–MS of blood-soluble EAdSc compounds. Network analysis revealed that the core targets include many inflammation-related targets, like MMP1/3/9, MAPK1/3/10/11, PPARG, IL2/6, RELA, PIK3CA, NLRP3, TLR4, and CASP1/3. GO and KEGG analysis also showed that EAdSc anti-UC effects are linked to several inflammation-related biological processes and multiple inflammatory signaling pathways. Among them, the NOD-like receptor signaling pathway was found to be closely related to the development of UC, and the TLR4/NF-κB/NLRP3 pathway is an important part of the NOD-like receptor signaling pathway [21]. It has been reported in the literature that *Sargentodoxa cuneata* can modulate the NF-κB/NLRP3 signaling pathway for the treatment of rheumatoid arthritis [22]. Therefore, we carried out the exploration of the mechanism of action of EAdSc against UC based on the TLR4/NF-κB/NLRP3 pathway. In addition, we also performed network pharmacological analyses of non-blood-soluble and blood-soluble EAdSc components, respectively. The results showed that the non-blood-soluble EAdSc components modulated the core targets TLR4, NF-κB p65, NLRP3, and CASP1 in the TLR4/NF-κB/NLRP3 pathway, whereas the blood-soluble EAdSc components only affected CASP1, suggesting that the non-blood-soluble EAdSc components play an important role in the anti-UC action of EAdSc (Figures S5 and S6). Therefore, we selected the non-blood-soluble EAdSc components and core targets in the TLR4/NF-κB/NLRP3 pathway for molecular docking analysis validation.

In this study, a classic DSS-induced UC animal model was used to evaluate the efficacy and underlying mechanism of action of EAdSc in UC. EAdSc significantly alleviated the DSS-induced clinical manifestations of UC, including diarrhea, bloody stool, and shortened colon length, in animals with UC. EAdSc treatment also reduced the heightened CMDI scores and enlarged spleens caused by DSS exposure. Previous clinical studies have found that the production of inflammatory factors, such as IL-1β, TNF-α, and IL-6, is significantly increased in the intestinal tract of patients with UC [23]. Notably, TNF-α is a dominant pathogenic cytokine associated with the pathogenesis of UC. Additionally, the regulatory effect of IL-6 on immunity is considered a strategic bridge between innate and adaptive immunity and is an ideal target for anti-inflammatory therapy [24]. In the body, IL-1β is mainly mediated by the activation of the NLRP3 inflammasome, which can trigger a cascade inflammatory response of the gut-acquired immune system [25]. In addition, Th17/Treg imbalance can disrupt the intestinal immune balance and cause damage to the intestinal mucosal barrier. Th17 cells are key effector T cells in the pathophysiology of UC that can promote the release of IL-6 and IL-17 and induce intestinal inflammatory injury [26,27]. In this work, EAdSc decreased the levels of IL-1β, IL-6, TNF-α, and IL-17 in the colon tissues of mice with UC. HE-stained histopathology also showed that EAdSc reduced the inflammatory infiltration of UC colon tissue, indicating the treatment had a definite treatment effect on UC.

It was found that compounds from EAdSc identified in the literature and by GC–MS, including eleutheroside A, liriodendrin, epicatechin, 2-methoxy-4-vinylphenol, catechin, androsin, coumaroyltyramine, and catechol, show high affinity to key proteins of the TLR4/NF-κB/NLRP3 pathway. Among these, salidroside, liriodendrin and epicatechin have been previously shown to have anti-UC effects, whereas 2-methoxy-4-vinylphenol and catechin have shown good anti-inflammatory activity [12,28,29,30,31]. These results suggest that EAdSc may treat UC through multicomponent interactions, which is consistent with the characteristics of most traditional Chinese medicines used in disease treatment. In addition, we found that EAdSc significantly decreased the mRNA expression of the key targets TLR4, NF-κB, NLRP3, and Caspase-1 of the TLR4/NF-κB/NLRP3 pathway, and the results of molecular docking and animal experiments somewhat validated the credibility of our network analysis results.

Our study had several limitations. First, we did not detect the constituents of EAdSc using LC–MS. Therefore, we could only perform a comparative analysis with the constituents of *Sargentodoxa cuneata* reported in the literature. Second, we applied the traditional method of decocting the *Sargentodoxa cuneata* and then extracting it to obtain the EAdSc. Therefore, the preparation of ethyl acetate extract of *Sargentodoxa cuneata* in the literature is slightly different from the method used in the present work, which may have caused differences in the final composition of the EAdSc obtained. In addition, the present work focused on the TLR4/NF-κB/NLRP3 pathway to verify the mechanism of EAdSc in treating UC. Future work can be performed on a wider range of possible pathways for an in-depth examination of the potential mechanisms. Moreover, direct evidence on whether EAdSc can exert therapeutic effects on UC by acting directly on colonic epithelial cells and modulating intestinal flora is lacking and can be explored in future work from a histological and intestinal flora perspective. Finally, in vivo experiments are required to confirm that the components entering the blood are effective components of EAdSc for the treatment of UC.

## 4. Materials and Methods

### 4.1. Chemicals and Reagents

Dextran sulfate sodium (DSS) (molecular weight 36–50 kDa) was obtained from MP Biomedical (Illkirch, France). Enteric-coated mesalazine tablets were purchased from Dr. Falk Pharma GmbH (Freiburg, Germany). Tween 80 was obtained from Xianshuigu Industrial Park (Tianjin, China). Formaldehyde was purchased from Chongqing Jiangchuan Chemical Group Co., Ltd. (Chongqing, China).

### 4.2. Plant Material and Preparation of EAdSc

*Sargentodoxa cuneata* was purchased from Taisheng Traditional Chinese Medicine Famous Products Market (Guiyang, China) and further identified by Wang, Guizhou Minzu University, China. The *Sargentodoxa cuneata* herbs were soaked in water for 15 min, heated to high heat until boiling, heated to low heat, decocted for 15 min, filtered, and concentrated using a rotary evaporator to obtain the extract. Appropriate amounts of water were used to dilute the extract, after which the extract was successively diluted with petroleum ether and ethyl acetate until colorless. The petroleum ether and ethyl acetate extraction solutions were concentrated using a rotary evaporator to obtain the petroleum ether extract and EAdSc.

### 4.3. Animals

Male Sprague-Dawley (SD) rats (6–8 weeks, 180–220 g) were obtained from Chongqing Tengxin Biological Technology Co., Ltd. (license number: SCXK-(Army) 2012-0011) (Chongqing, China), and male Kunming (KM) mice (6–8 weeks, 18–22 g) were obtained from Speifu (Beijing, China) Biotechnology Co., Ltd. (license number: SCXK (Beijing, China) 2019-0010). All animals received a standard diet and water. Mice and rats were housed in a controlled room with a 12 h light/12 h dark cycle at 24 ± 2 °C temperature and 55–70% humidity. Animal studies were approved by the Animal Experiment Ethics Committee of Guizhou University of Traditional Chinese Medicine [No. 20210159] and [No. 20220003].

### 4.4. Chemical Profiling of EAdSc

#### 4.4.1. Identification of EAdSc Components by GC–MS

Gas chromatography–mass spectrometry (GC–MS) was performed on an Agilent HP6890/5975C gas chromatograph with an Agilent HP-5MS mass spectrometer (60 m × 0.25 mm × 0.25 μm). An elastic quartz capillary column was used for chromatographic separation. The programmed oven temperatures were as follows: initial temperature at 40 °C for 2 min, increased to 215 °C at a heating rate of 3.5 °C/min, and then increased to 310 °C at a heating rate of 8 °C/min. The total analysis time was 74 min. The gasification chamber temperature was 230 °C, and the carrier gas was 1.0 mL/min helium. The temperature of the ion source was set at 230 °C, and the mass scanning range was set between 29 and 500 amu in the full scan mode. The injection was performed in the split mode with a split ratio of 10:1. Compounds were identified by comparing the obtained mass spectra of the analytes with those of authentic standards from the NIST 17 library and Wiley 275 standard mass spectra. The relative content of each chemical compound was calculated using the peak area normalization method.

#### 4.4.2. Chemical Profiling of Plasma Obtained after EAdSc Administration

Ten rats were intragastrically administered EAdSc (200 mg/kg) for 3 days. At 15 min, 30 min, 1 h, 2 h, and 4 h after the last administration, two rats were anesthetized and sacrificed at each time point, and blood was collected through their femoral arteries. The collected blood was anticoagulated using sodium heparin. The samples were centrifuged at 10,000 rpm for 10 min at 4 °C, and the resulting supernatant was considered the drug-containing plasma. The drug-containing sera obtained at each of the five time points were mixed, and 5 mL of the mixed sera was added to 50 mL of extract liquor (methanol), vortexed for 2 min, and centrifuged at 13,000 rpm for 10 min. The resulting supernatant was evaporated until dry, dissolved into 500 μL methanol, and centrifuged once again at 13,000 rpm for 10 min again. The supernatant was used for LC–MS analysis. Additionally, a control group was established. Except for the administration of EAdSc, all procedures were the same as those for the administration group.

The blood-soluble components of the EAdSc were identified. Briefly, a SHIMADZU Nexera UPLC LC-3A system (Kyoto, Japan) and an AB SCIEX^TM^ Triple TOF5600^+^ mass spectrometer were used for analysis. Elution was performed in a Waters BEH C_18_ (150 mm × 2.1 mm, 2.5 µm) with a column temperature of 40 °C and a constant flow rate of 0.3 mL/min. The injection volume was 10 µL for each determination. The elution program was as follows: 0–2 min, 5–5% B; 2–10 min, 5–95% B; 10–13 min, 95–95% B; and 13–15 min, 95–5% B (solvent A, 0.1% formic acid; solvent B, acetonitrile). Spectra were acquired in both positive and negative ionization modes, with the capillary voltage set at −4400 V and +5500 V. The collected data were further processed using MS-DIAL 4.60 software and searched qualitatively through MassBank, Respect, and the GNPS library.

### 4.5. Network Pharmacology Analysis

The NCBI PubChem Compound (https://www.ncbi.nlm.nih.gov/pccompound/, accessed on 14 September 2022) and Swiss Target Prediction platforms (http://www.swisstargetprediction.ch/, accessed on 3 August 2023) were used to identify the potential targets of the EAdSc components. Gene data were obtained from the GSE206285 gene chip test results in the GEO database (https://www.ncbi.nlm.nih.gov/geo/, accessed on 11 October 2022), and the absolute values of the gene difference between healthy subjects and UC patients (|log FC|) ≥ 1, and *p* < 1 × 10^−15^ were used as screening criteria to analyze the gene expression information and obtain the differential genes of UC. Using EAdSc components, EAdSc component targets, and UC targets combined with the STRING platform (https://cn.string-db.org/, accessed on 3 August 2023), the “component–target–disease” network of EAdSc components was constructed by Cytoscape 3.9.1. The maximal clique centrality (MCC), maximum neighborhood component (MNC), Closeness Centrality, Betweenness Centrality, and Degree algorithms in the plug-in CytoHubba were used to screen core targets, and the targets that simultaneously satisfied greater than the median of the above five parameters were considered core targets.

To comprehensively evaluate the action network of the EAdSc components, we performed Gene Ontology (GO) and Kyoto Encyclopedia of Genes and Genomes (KEGG) analyses on core targets of EAdSc components and UC. Using the R language, the data package “org.Hs.eg.db” was used to encode the gene. Then, the data packages “colorspace” and “stringi,” as well as the “DOSE,” “clusterProfiler,” and “pathview” data packages of the Bioconductor platform and the ClueGo plug-in in the Cytoscape 3.9.1 software were used to perform GO biological process enrichment analysis and KEGG pathway enrichment analysis.

### 4.6. Molecular Docking

Molecular docking technology was used to identify the components of EAdSc effective against UC based on the TLR4/NF-κB/NLRP3 pathway. The core non-blood-soluble EAdSc compounds (*mol2 format) were downloaded from the Traditional Chinese Medicines Systems Pharmacology (TCMSP) database, while the 3D structures (*PDB format) of the various target proteins, TLR4 (PDB:3FXI), NF-κB p65 (PDB:7O6J), NLRP3 (PDB:7ALV), ASC (PDB:5H8O) and Caspase-1 (PDB:6F6R) were obtained from the Protein Data Bank (PDB) database (https://www.rcsb.org/, accessed on 5 August 2023). The core protein PyMol software 2.6 was used to virtually perform dehydration and hydrogenation of the proteins, and the format of the core compounds and target proteins was converted to *pdbqt format using AutoDock software 4.2. Docking analysis was performed using AutoDock Vina software. The grid boxes for TLR4 (box coordinates: 4.61, −5.302, 24.862; box size: 60, 60, 84), NF-κB p65 (box coordinates: −19.058, −12.156, 7.438; box size: 40, 40, 40), NLRP3 (box coordinates: 19.798, 33.787, 132.561; box size: 52, 50, 50), ASC (box coordinates: −4.674, 7.713, 24.386; box size: 56, 40, 52), and Caspase-1 (box coordinates: 12.524, 30.44, −6.638; box size: 52, 54, 54) Å were applied. The docking results were visualized using Discovery Studio Visualizer software 2016. The protein–ligand complex with the lowest binding energy (kcal/mol) was determined to be the most suitable structure.

### 4.7. Pharmacodynamic Evaluation and Validation of the Mechanism of EAdSc for the Treatment of UC

#### 4.7.1. Induction of UC in Mice and Subsequent Treatment

After acclimatization for 7 days, the mice were randomly assigned to one of six groups (n = 8 per group): control group, UC group, mesalazine (0.52 g/kg) group, and three EAdSc groups of varying concentrations (14.63 mg/kg, 29.25 mg/kg, 58.50 mg/kg). The mesalazine and EAdSc doses were determined based on the clinical doses and yield of the extract. The 14.63 mg/kg dose of EAdSc was equivalent to a human clinical dose of 0.25 g/kg of *Sargentodoxa cuneata*. UC was induced in mice by administration of 3.0% (*w*/*v*) DSS in drinking water for 7 days. Mice were then given normal drinking water for another 3 days before sacrifice. From the first day of giving DSS in drinking water, the control and UC groups were given free water, the mesalazine group was administered 0.52 g/kg mesalazine by gavage, and the EAdSc groups were dosed by intragastric gavage with corresponding oral doses of EAdSc daily for 10 days. On Day 10, the mice were sacrificed by cervical dislocation, and the lengths of the colon tissue from the proximal end of the rectum to the ileocecal junction were measured. Simultaneously, the spleen was weighed, and the spleen index was calculated using the following formula: Spleen index (100%) = spleen weight/mouse body weight × 100. Colon tissues were immobilized in 4% paraformaldehyde solution or stored at −80 °C for further analyses.

#### 4.7.2. Disease Activity Index (DAI) and Colon Macroscopic Damage Index (CMDI)

Body weight, stool consistency, and occult blood levels were recorded daily during UC induction. The DAI was calculated as described in a previous study using the following formula: DAI = (weight loss score + fecal shape score + bloody stool score)/3 [32]. The CMDI was calculated as follows: 0 points, normal; 1 point, mild congestion, edema, smooth surface, and five erosions or ulcers; 2 points, moderate congestion, edema, erosion, and no ulcers; 3 points, high congestion and edema, mucosal surface necrosis and ulceration, and the main part of the damage along the colon extends <1 cm; and 4 points, severe congestion and edema, mucosal necrosis and ulceration, and the main part of the damage along the colon extends >1 cm [33].

#### 4.7.3. Histological Examination

Colon tissues were fixed in 4% paraformaldehyde, and the hematoxylin and eosin (H&E) stain was performed, and histopathological changes were observed and evaluated in a blinded fashion, as previously described [33]. The histological damage index (TDI) was scored by combining the depth of the ulcer, degree of inflammatory cell infiltration, and depth of inflammatory cell infiltration as follows: 0 points, normal; 1 point, epithelial ulceration, mild inflammatory cell infiltration, and mucosal inflammatory cell infiltration; 2 points, mucosal lamina propria ulceration, severe inflammatory cell infiltration, and mucosal and submucosal inflammatory cell infiltration; 3 points, muscularis mucosal ulceration, severe inflammatory cell infiltration, and inflammatory cell infiltration of the entire colon wall.

#### 4.7.4. ELISA Analysis

The supernatants of the subsequently homogenized colon tissues were obtained to detect production of the pro-inflammatory factors IL-1β, TNF-α, IL-6, and IL-17 using corresponding ELISA kits (Shanghai Zhuocai Biotechnology Co., Ltd., Shanghai, China) according to the manufacturer’s instructions.

#### 4.7.5. Experimental Validation of RT-PCR Analysis

For RT-PCR analysis, total cellular RNA was isolated from the colon tissue using RNA TRIzol Reagent (Hefei Bomei Biotechnology Co., Ltd., Hefei, China), and cDNA was synthesized from the resulting total RNA using the PrimeScript RT Reagent Kit (Baoriyi Biotechnology Co., Ltd., Beijing, China). The amplification reactions were conducted in 96-well reaction plates with a 20 μL reaction volume (Baoriyi Biotechnology Co., Ltd., Beijing, China). In this study, the following primer sequences were used: NLRP3 F: 5′-CCAGATGGAGAAGGCAGATCACTTG-3′, R: 5′-TCGCAGCAAAGATCCACACAGC-3′; p65 F: 5′-CCAAGACACACCCCACCATCAAG-3′, R: 5′-AGGTCAGCCTCATAGTAGCCATCC-3′; Caspase-1 F: 5′-AGAGGATTTCTTAACGGATGCA-3′, R: 5′-TCACAAGACCAGGCATATTCTT-3′; TLR4 F: 5′-GCCATCATTATGAGTGCCAATT-3′, R: 5′-AGGGATAAGAACGCTGAGAATT-3′; and ACTB F: 5′-CATCACTATTGGCAACGAGCGGTTCC-3′, R: 5′-ACGCAGCTCAGTAACAGTCCGCCTA-3′. The fold change in the level of the target mRNA between the control and treated animal groups was corrected to the level of ACTB. Finally, the change in gene expression was calculated by 2^−ΔΔCt^.

### 4.8. Statistical Analysis

Data are presented as mean ± standard error of the mean (SEM). Using SPSS software (version 20.0; SPSS Inc., Chicago, IL, USA), the means were assessed using one-way analysis of variance (ANOVA), and the Fisher’s protected least significant difference (LSD) post hoc test was used to determine the significance for multiple comparisons. *p* < 0.05 was considered statistically significant.

## 5. Conclusions

In this work, 29 compounds were identified in EAdSc using GC–MS, and 22 blood-soluble EAdSc components were recognized. Network pharmacology analysis revealed that multiple inflammatory signaling pathways are involved in EAdSc anti-UC activity. Furthermore, molecular docking analysis found that eleutheroside A, liriodendrin, epicatechin, 2-methoxy-4-vinylphenol, catechin, androsin, coumarouyltyramine, and catechol may be the active components of EAdSc in the treatment of UC through the TLR4/NF-κB/NLRP3 signaling pathway. In addition, we first demonstrated that EAdSc significantly improved the DSS-induced clinical symptoms and intestinal inflammatory injury of mice with UC. EAdSc could regulate the TLR4/NF-κB/NLRP3 signaling pathway and decreasing the pro-inflammatory cytokine levels in colon tissues. This study provides a basis for the use of EAdSc in the treatment of UC and provides support for determining the mechanisms of action of various active constituents.

## Figures and Tables

**Table 1:** The results of GC–MS analysis of the ethyl acetate extract from decoction of *Sargentodoxa cuneata* (EAdSc).

Retention Time (min)	Compound	Molecular Weight	Percentage (%)
7.753	Propanoic acid, ethyl ester	102.07	2.441938
9.485	Toluene	92.06	0.119818
10.662	Hexanal	100.09	0.209041
11.059	Acetic acid, butyl ester	116.08	6.236906
13.052	Ethylbenzene	106.08	0.097175
13.381	p-Xylene	106.08	0.234868
14.336	Cyclohexanone	98.07	4.841384
25.542	2,3-Dihydro-3,5-dihydroxy-6-methyl-4H-Pyran-4-one	144.04	10.60658
26.783	dihydro-4-hydroxy-2(3H)-Furanone	102.03	0.305965
26.87	Benzoic acid	122.04	0.772141
27.55	3,5-Dihydroxy-2-methyl-4H-Pyran-4-one	142.03	1.080466
28.758	Catechol	110.04	4.13385
31.517	Hydroquinone	110.04	0.778671
32.552	2-Methoxy-4-vinylphenol	150.07	2.0374
33.993	2,6-Dimethoxy-phenol	154.06	0.500634
34.193	Eugenol	164.08	0.290604
36.913	4-Hydroxy-benzeneethanol	138.07	9.224397
39.02	Apocynin	166.06	3.120635
41.609	4-Ethenyl-2,6-dimethoxy-phenol	180.08	0.470446
41.849	Vanillic acid	168.04	2.444373
43.116	3,4,5-Trimethoxy-phenol	184.07	0.866043
47.176	1-(4-Hydroxy-3,5-dimethoxyphenyl)-Ethanone	196.07	0.199561
49.71	4-Hydroxy-3,5-dimethoxy-benzoic acid	198.05	0.369855
53.374	n-Hexadecanoic acid	256.24	0.721303
56.987	9,12-Octadecadienoic acid (Z, Z)-	280.24	0.543
57.117	9,12,15-Octadecatrienoic acid, (Z, Z, Z)-	278.23	0.765977
57.451	Octadecanoic acid	284.27	0.068583
66.239	Octacosane	394.45	0.338781
68.751	Triacontane	422	0.144914

**Table 2:** The potential components of the ethyl acetate extract from decoction of *Sargentodoxa cuneata* (EAdSc) for molecular docking analysis.

Compounds	TLR4	p65	NLRP3	ASC	Caspase-1
2,3-Dihydro-3,5-dihydroxy-6-methyl-4H-pyran-4-one	−5.1	−4.5	−5.4	−4.6	−4.6
2-Methoxy-4-vinylphenol	−7.3	−6.9	−9	−7	−7.1
Apocynin	−5.6	−4.9	−6.2	−5.6	−5.1
Benzoic acid	−5.6	−4.5	−5.8	−5.3	−5
Catechol	−7.5	−6.8	−8.8	−6.8	−6.8
Cedar acid	−5.3	−5.1	−5.9	−5.4	−5.1
Eugenol	−5.9	−5.2	−6.2	−6.1	−5.5
Hydroquinone	−4.7	−4.2	−5.3	−4.3	−4.6
Linoelaidic acid	−7	−4.9	−6.5	−5.5	−5
Linolenic acid	−6.9	−4.9	−6.7	−5.1	−5.6
Octadecanoic acid	−6.5	−4.6	−5.9	−4.7	−4.8
Palmitic acid	−6.2	−4.5	−5.9	−5.3	−4.9
Tyrosol	−5.4	−4.5	−5.9	−5.2	−4.9
Vanillic acid	−5.4	−4.8	−6.2	−5.5	−5.1
2-(1-Methylethenyl)-5-benzofurancarboxylic acid [15]	−7.2	−6	−7.4	−7.4	−5.9
2-Hydroxy-succinic acid-1-methylester [15]	−4.5	−4.2	−4.9	−4.2	−4.2
3,4-Dihydroxyphenylethyl alcohol glucoside [15]	−7.2	−6.1	−7.8	−6.5	−6.5
3,5-O-Dimethylgallic acid [16]	−5.7	−5.4	−6.5	−5.6	−5.4
5-Hydroxymaltol [15]	−5.1	−4.5	−5.5	−4.8	−5.1
Androsin [15]	−6.6	−6.2	−6.9	−8.1	−6
Caffeic acid [15]	−5.9	−5.4	−6.8	−5.9	−5.7
Catechin [15]	−7.5	−6.8	−8.8	−6.8	−6.8
Chlorogenic acid [16]	−7.1	−6.6	−8.4	−6.9	−6.8
Coumaroyltyramine [15]	−7.6	−6.3	−7.6	−7.1	−7.2
Eleutheroside A [15]	−9.9	−6.9	−9	−8.2	−7
Epicatechin [15]	−7.5	−7	−8.4	−7.1	−6.5
Ethyl 3,4-dihydroxybenzoate [15]	−5.8	−5.3	−6.3	−5.8	−5.2
Ferulic acid [15]	−6	−5.6	−6.6	−6.5	−5.2
Glucosyringic acid [15]	−6.8	−6	−6.8	−6.2	−6.4
Hydroxytyrosol [15]	−5.7	−5.1	−6.2	−5.6	−5.2
Liriodendrin [15]	−8.5	−6.7	−8.4	−7.8	−7.3
Methyl caffeate [15]	−6	−5.7	−6.6	−5.9	−5.3
Methyl p-coumarate [15]	−6.3	−5.7	−6.4	−6.2	−5.5
Methyl protocatechuate [15]	−5.6	−4.8	−6.2	−5.6	−5.1
N-(p-Hydroxyphenethyl) ferulamideamide [16]	−7.5	−6.4	−8.3	−6.8	−7
p-Hydroxybenzoic acid [15]	−5.4	−5.1	−5.9	−5.3	−5
Protocatechuic acid [15]	−5.7	−5.3	−6.4	−6.1	−5.1
Salidroside [15]	−6.8	−5.9	−7.9	−6.4	−6.1

**Table 3:** The blood-dissolved components of the ethyl acetate extract from decoction of *Sargentodoxa cuneata* (EAdSc).

Components	Retention Time (min)	Precursor *m*/*z*	Adduct	Reference *m*/*z*	Total Score
[(1S,2R,4S,5R,9R,10R,14S,15S,17S)-9-(Furan-3-yl)-1-hydroxy-15-[(1R)-1-hydroxy-2-methoxy-2-oxoethyl]-10,14,16,16-tetramethyl-7,18-dioxo-3,8-dioxapentacyclo[12.3.1.02,4.04,13.05,10]octadecan-17-yl]propanoate	6.891567	592.279	[M+H]^+^	592.27521	95
2-[(4-Ethyl-8,8-dimethyl-2-oxo-9,10-dihydropyrano[2,3-h]chromen-5-yl)oxy]-N-(furan-2-ylmethyl)acetamide	6.971817	412.1805	[M+H]^+^	412.17599	90.3
4-[[[2-[(8,8-Dimethyl-2-oxo-4-propyl-9,10-dihydropyrano[2,3-h]chromen-5-yl)oxy]acetyl]amino]methyl]cyclohexane-1-carboxylic acid	7.51045	486.2485	[M+H]^+^	486.24899	99.9
Tripterifordin	8.068583	319.2198	[M+H]^+^	319.22	100
(9Z,12E)-15,16-Dihydroxyoctadeca-9,12-dienoic acid	8.240916	335.2125	[M+H]^+^	335.2157	94.9
N-[(2S)-1-[(2-Amino-2-oxoethyl)amino]-4-methyl-1-oxopentan-2-yl]-1-[1-(4-methylphenyl)sulfonylpiperidine-4-carbonyl]pyrrolidine-2-carboxamide	8.729733	550.2736	[M+H]^+^	550.27002	94.8
(4S,5Z,6S)-4-(2-Methoxy-2-oxoethyl)-5-[2-[(E)-3-phenylprop-2-enoyl]oxyethylidene]-6-[(2S,3R,4S,5S,6R)-3,4,5-trihydroxy-6-(hydroxymethyl)oxan-2-yl]oxy-4H-pyran-3-carboxylic acid	9.1427	342.1837	[M+H]^+^	342.18704	94.6
Methyl (4R,8aS)-1-hydroxy-2-(hydroxymethyl)-5,5,8a-trimethyl-4-[(2E,4E,6E)-octa-2,4,6-trienoyl]oxy-4a,6,7,8-tetrahydro-4H-naphthalene-1-carboxylate	10.51628	457.2004	[M+H]^+^	457.19867	98.4
Sibiromycin-494 hemiaminal	10.51628	494.2882	[M+H]^+^	494.28601	97.7
[(1R,5R,9S,13S)-5,9,13-Trimethyl-5-tetracyclo[11.2.1.01,10.04,9]hexadec-14-enyl]methanol	10.51628	311.2368	[M+H]^+^	311.23453	97.3
(2S,3R,4S,5S,6R)-2-[(2R,3R,4S,5S,6R)-4,5-Dihydroxy-6-(hydroxymethyl)-2-[[(3S,8R,10R,12R,14R,17S)-12-hydroxy-4,4,8,10,14-pentamethyl-17-[(2S)-6-methyl-2-[(2S,3R,4S,5S,6R)-3,4,5-trihydroxy-6-[[(2S,3R,4S,5S)-3,4,5-trihydroxyoxan-2-yl]oxymethyl]oxan-2-yl]oxyhept-5-en-2-yl]-2,3,5,6,7,9,11,12,13,15,16,17-dodecahydro-1H-cyclopenta[a]phenanthren-3-yl]oxy]oxan-3-yl]oxy-6-(hydroxymethyl)oxane-3,4,5-triol	10.63595	1061.585	[M+H]^+^	1061.58997	88.2
4-Methylabyssinone V	10.7956	445.197	[M+H]^+^	445.19852	98.8
(2Z,6E,10Z)-12-Acetyloxy-10-(acetyloxymethyl)-6-methyl-2-(4-methylpent-3-enyl)dodeca-2,6,10-trienoic acid	11.4014	459.2161	[M+H]^+^	459.22003	92.5
Isosafrole	11.44157	313.2502	[M+H]^+^	313.25	100
(E)-3-(4-Methoxyphenyl)-1-[2,4,6-trimethoxy-3-(3-methylbut-2-enyl)phenyl]prop-2-en-1-one	11.48123	419.2229	[M+H]^+^	419.22476	98.2
[(2R)-2-[(E,2S,4R)-4,6-Dimethyloct-6-en-2-yl]-6-oxo-2,3-dihydropyran-3-yl] (2E,4E,6S)-8-hydroxy-6-(hydroxymethyl)-4-methylocta-2,4-dienoate	12.08187	473.2306	[M+H]^+^	473.22998	99.8
(1R,7R)-7-Ethenyl-1,4a,7-trimethyl-3,4,4b,5,6,9,10,10a-octahydro-2H-phenanthrene-1-carboxylic acid	12.69688	301.246	[M-H]^-^	301.24362	97.2
(3S,4S,6aR,6bS,8R,8aR,12aS,14bR)-8-Hydroxy-4,6a,6b,11,11,14b-hexamethyl-3-[(2S,3R,4S,5R)-3,4,5-trihydroxyoxan-2-yl]oxy-1,2,3,4a,5,6,7,8,9,10,12,12a,14,14a-tetradecahydropicene-4,8a-dicarboxylic acid	13.61262	657.3256	[M+H]^+^	657.3252	100
[1,3,12-Triacetyloxy-17-(furan-3-yl)-4,4,8,10,13-pentamethyl-2,3,5,6,7,9,11,12,16,17-decahydro-1H-cyclopenta[a]phenanthren-7-yl] 2-hydroxy-3-methylpentanoate	14.05742	688.4067	[M+H]^+^	688.40601	99.9
(3S,10R,13R)-10,13-Dimethyl-17-octyl-2,3,4,7,8,9,10,11,12,13,14,15,16,17-tetradecahydro-1H-cyclopenta[a]phenanthren-3-yl (4-nitrophenyl) carbonate	14.09792	552.3605	[M+H]^+^	552.35999	99.9
1,2,6b,9,9,12a-Hexamethyl-4a-[3,4,5-trihydroxy-6-(hydroxymethyl)oxan-2-yl]oxycarbonyl-10-(3,4,5-trihydroxy-6-methyloxan-2-yl)oxy-2,3,4,5,6,6a,7,8,8a,10,11,12,13,14b-tetradecahydro-1H-picene-6a-carboxylic acid	14.38025	812.4857	[M+H]^+^	812.47906	92
1,2-Dihydroxyheptadec-16-yn-4-yl acetate	14.39328	325.2124	[M-H]^−^	325.21054	98.3

## Data Availability

Data are contained within the article.

## Supplementary Materials

The following supporting information can be downloaded at: https://www.mdpi.com/article/10.3390/molecules28227663/s1, Figure S1. The total ion chromatogram of the ethyl acetate extracts from decoction of *Sargentodoxa cuneata* (EAdSc). Figure S2. The MS spectra of control serum and drug-containing serum. (a) The mass spectra of control serum sample in positive ion mode. (b) The mass spectra of control serum sample in negative ion mode. (c) The mass spectra of drug-containing serum sample of EAdSc in positive ion mode. (d) The mass spectra of drug-containing serum sample of EAdSc in negative ion mode. Figure S3. The “component–target–disease” interaction network of the ethyl acetate extracts from decoction of *Sargentodoxa cuneata* (EAdSc). Orange circles represent targets, blue triangles represent compounds, and red diamonds represent UC. Figure S4. The affinity of potential compounds in the ethyl acetate extracts from decoction of *Sargentodoxa cuneata* (EAdSc) with the core targets of the TLR4/NF-κB/NLRP3 pathway. Figure S5. Network pharmacological analysis of the anti-ulcerative colitis effects of the non-blood-soluble EAdSc components. (a) “Drug–target–disease” network, (b) protein–protein interaction network for intersection targets and clustering results, and (c) the core targets. Figure S6. Network pharmacological analysis of the anti-ulcerative colitis effects of the blood-soluble EAdSc components. (a) “Drug–target–disease” network, (b) protein–protein interaction network for intersection targets and clustering results, and (c) the core targets. Table S1. The components with a total score higher than 80 in the control serum. Table S2. The components with a total score higher than 80 in the drug-containing serum of EAdSc. Table S3. The core targets of EAdSc components against UC.
