# Supplementary material for: Anti-Ulcerative Colitis Effects and Active Ingredients in Ethyl Acetate Extract from Decoction of Sargentodoxa cuneata
Source: Molecules. 2023 Nov 19;28(22):7663. doi: 10.3390/molecules28227663 (PMC10675221; doi:10.3390/molecules28227663)
Supplement: Supplementary file 1 [file molecules-28-07663-s001.zip › molecules-2654920-supplementary.pdf]

Supplementary Material

Supplementary Figures and Tables

Supplementary Figures

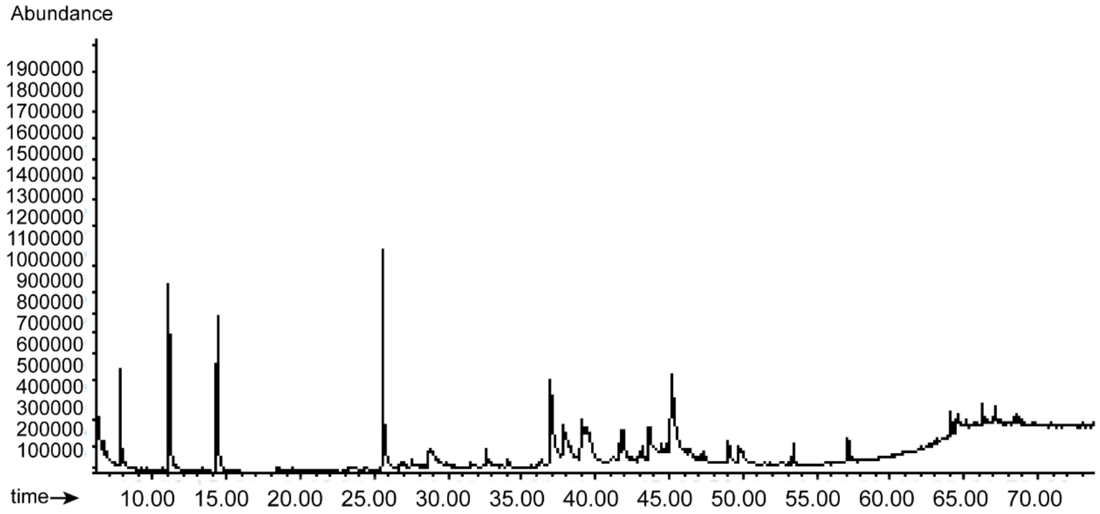

Supplementary Figure S1. The total ion chromatogram of the ethyl acetate extracts from decoction of *Sargentodoxa cuneata* (EAdSc).

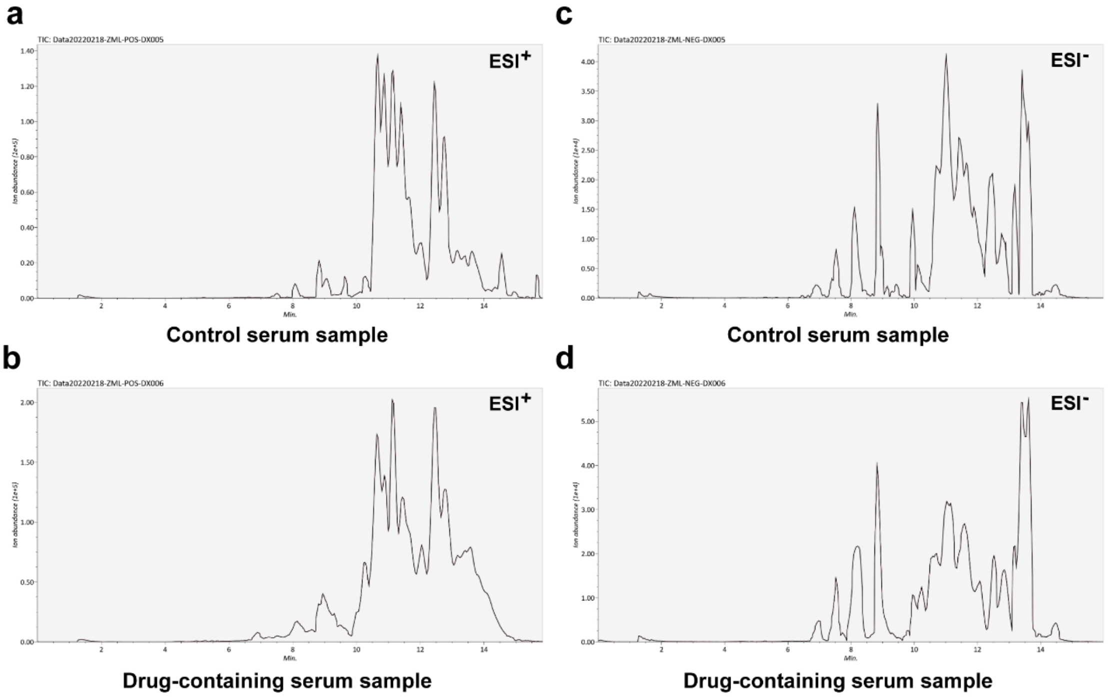

Supplementary Figure S2. The MS spectra of control serum and drug-containing serum. (**a**) The mass spectra of control serum sample in positive ion mode. (**b**) The mass spectra of control serum sample in negative ion mode. (**c**) The mass spectra of drug-containing serum sample of EAdSc in positive ion mode. (**d**) The mass spectra of drug-containing serum sample of EAdSc in negative ion mode.

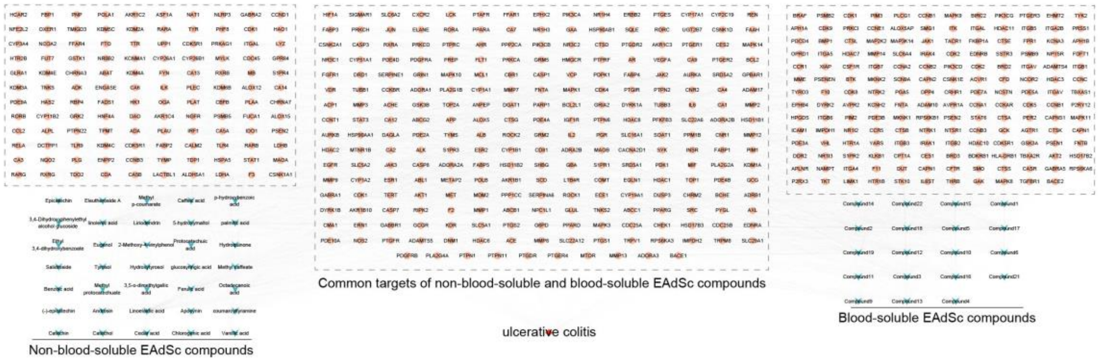

Supplementary Figure S3. The "component–target–disease" interaction network of the ethyl acetate extracts from decoction of *Sargentodoxa cuneata* (EAdSc). Orange circles represent targets, blue triangles represent compounds, and red diamonds represent UC.

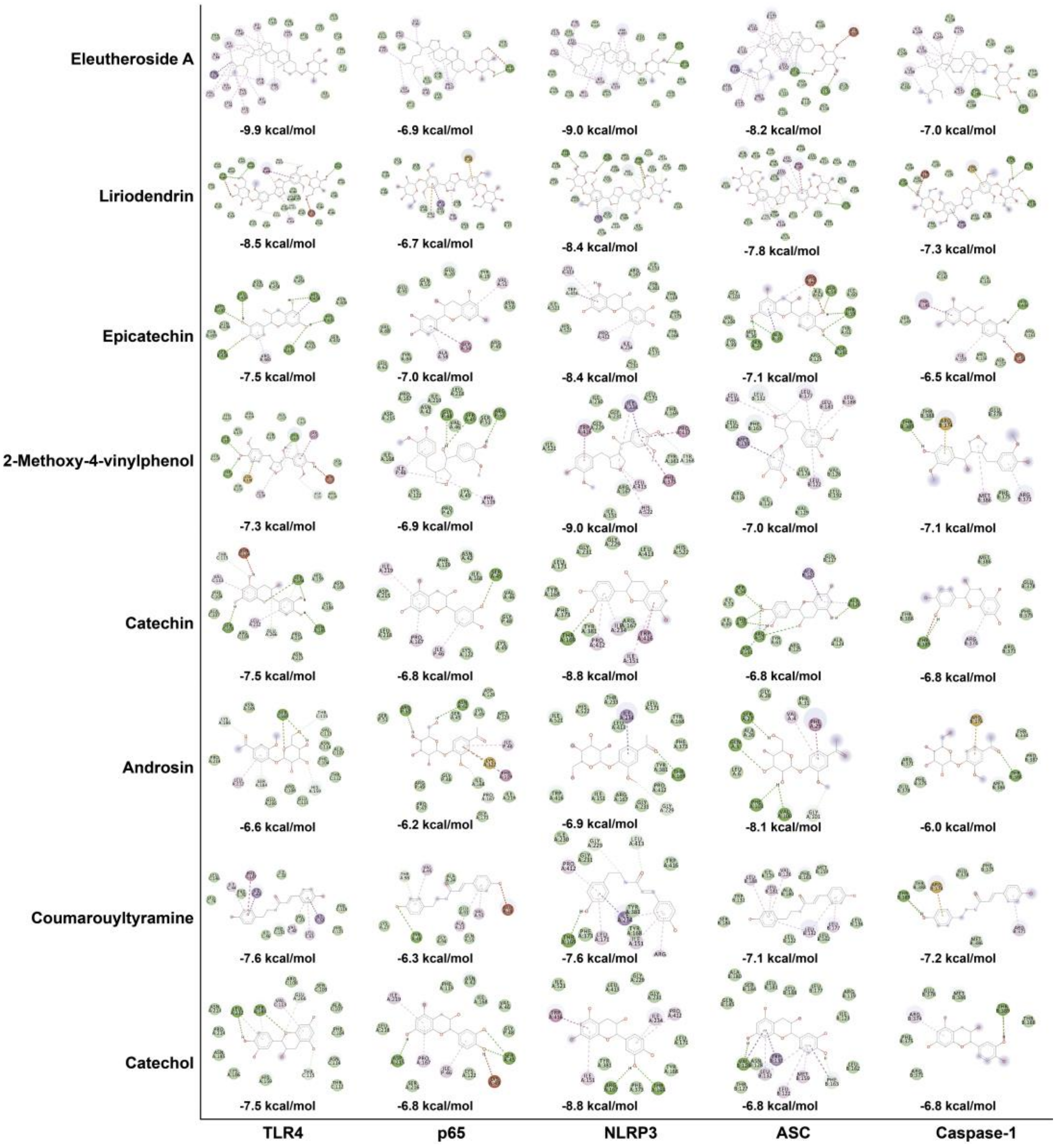

Supplementary Figure S4. The affinity of potential compounds in the ethyl acetate extracts from decoction of *Sargentodoxa cuneata* (EAdSc) with the core targets of the TLR4/NF-κB/NLRP3 pathway.

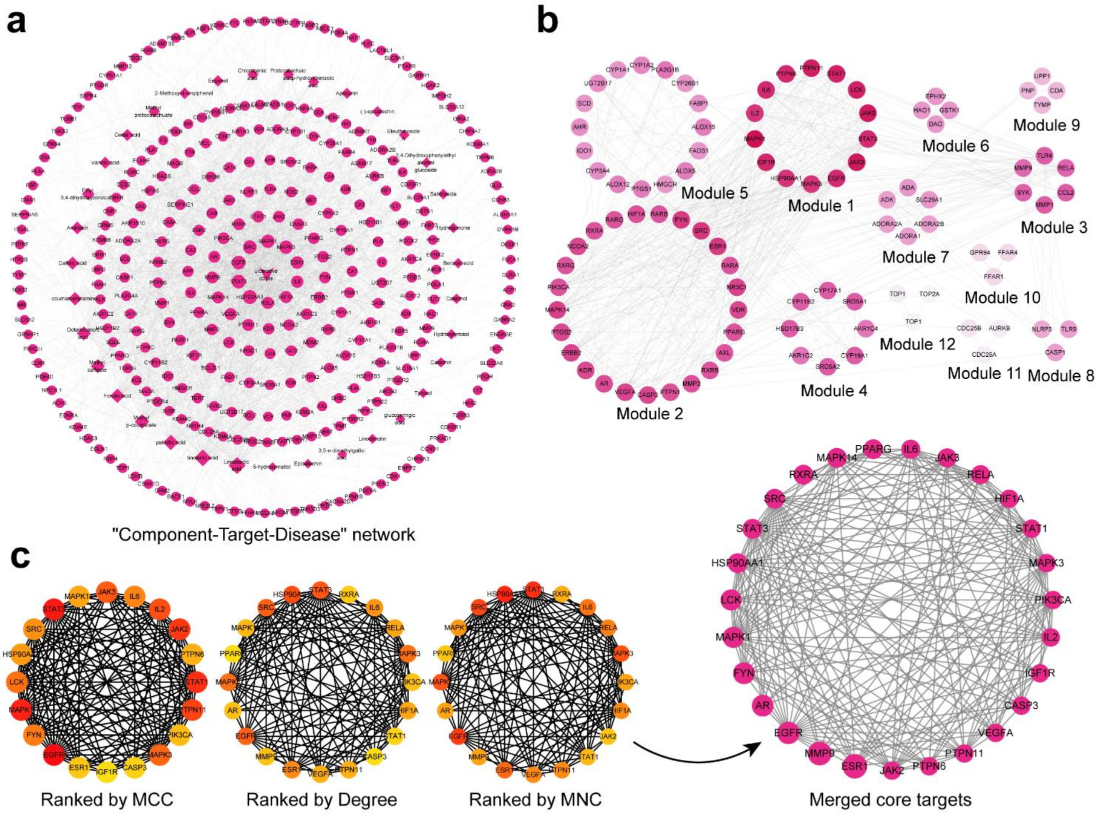

Supplementary Figure S5. Network pharmacological analysis of the anti-ulcerative colitis effects of the non-blood-soluble EAdSc components. (**a**) "Drug–target–disease" network, (**b**) protein-protein interaction network for intersection targets and clustering results, and (**c**) the core targets.

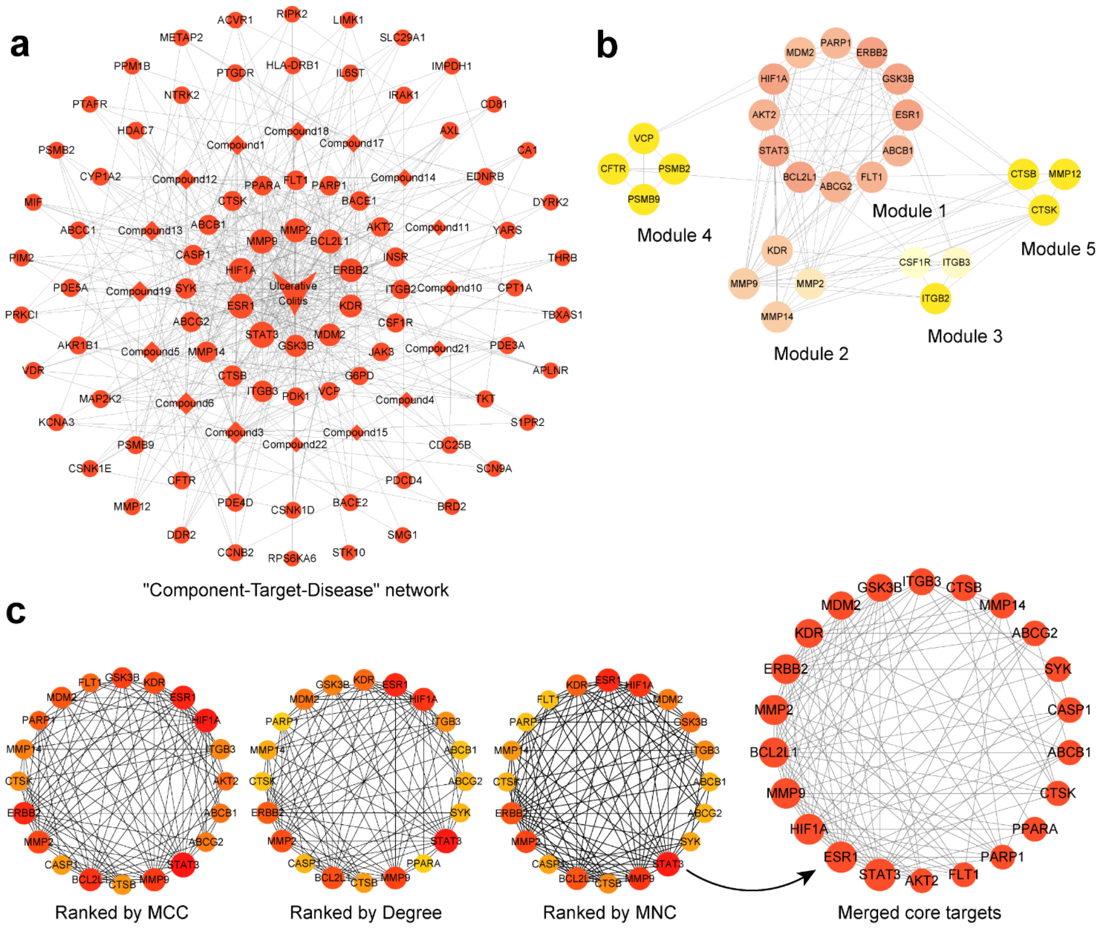

Supplementary Figure S6. Network pharmacological analysis of the anti-ulcerative colitis effects of the blood-soluble EAdSc components. (**a**) "Drug–target–disease" network, (**b**) protein-protein interaction network for intersection targets and clustering results, and (**c**) the core targets.

Supplementary Tables

Supplementary Table S1. The components with a total score higher than 80 in the control serum.

Title	RT (min)	Precursor m/z	Adduct	Reference m/z	Formula	Ontology	Total score
2-(2-(2,5-dioxohexahydroimidazo[4,5-d]imidazol-1(2H)-yl)acetamido)acetic acid	1.31305	280.0699	[M+H]+	280.07	C8H11N5O5	N-acyl-alpha amino acids	100
Eicosanoids_12,13diHOME_C18H34O4	8.069867	337.2284	[M+H]+	337.235			80.3
2-(7-hydroxy-6-methyloctyl)-2H-furan-5-one	8.825817	227.1629	[M+H]+	227.164	C13H22O3	Fatty alcohols	99.4
Dihydrojasmonic Acid	8.825817	213.1472	[M+H]+	213.1485	C12H20O3	Jasmonic acids	99.2
methyl (2E,8E)-9-[3a-hydroxy-6,7-dimethyl-1-(2-methylpropyl)-3-oxo-2,4,7,7a-tetrahydro-1H-isoindol-4-yl]-4,5-dihydroxy-8-methylnona-2,8-dienoate	8.95665	472.271	[M+H]+	472.2721	C25H39NO6	Isoindolones	99.4
4-PYRIDOXATE	8.95665	184.0587	[M+H]+	184.06	C8H9NO4	Pyridinecarboxylic acids	99.1
Fenpropidin	9.036966	274.2529	[M+H]+	274.2529	C19H31N	Phenylpropanes	100
Amorolfine Hydrochloride	9.077133	318.2789	[M+H]+	318.279	C21H36ClNO	Phenylpropanes	100
3-[3-[3,4-dihydroxy-5-(6-oxo-3H-purin-9-yl)oxolan-2-yl]propanoyl]benzoic acid	9.626884	437.162	[M+H]+	437.162	C19H18N4O7	5'-deoxyribonucleosides	100
n trans p coumaroyltyramine	10.02542	284.2746	[M+H]+	284.279	C17H17NO3	Coumaric acids and derivatives	90.9
3-(2,4-dihydroxyphenyl)-7-hydroxy-6,8-bis(3-methylbut-2-enyl)-2,3-dihydrochromen-4-one	10.2001	431.1875	[M+H]+	431.1851	C25H28O5	8-prenylated isoflavanones	97.1
(1R,2R,4aS,6aS,6bR,9R,10R,11R,12aR)-1,10,11-trihydroxy-9-(hydroxymethyl)-1,2,6a,6b,9,12a-hexamethyl-2,3,4,5,6,6a,7,8,8a,10,11,12,13,14b-tetradecahydropicene-4a-carboxylic acid	10.28575	505.1956	[M+H]+	505.191	C30H48O6	Triterpenoids	90.2
methyl 8-hydroxy-4,5,7,10,14,14-hexamethyl-6,17-dioxo-16-	10.37117	481.1977	[M+H]+	481.1987	C26H34O6	Naphthopyrans	99.6

oxapentacyclo[13.2.2.0?,??.0?,??.0?,?]nonadeca-3,7-diene-9-carboxylate							
6-[(3E,6E)-2,5-dihydroxy-4,6-dimethyl-7-(1,2,4-trimethyl-3,6-dioxabicyclo[3.1.0]hexan-4-yl)hepta-3,6-dien-2-yl]-4-methoxy-3,5-dimethylpyran-2-one	10.50507	457.2011	[M+H]+	457.203	C24H34O7	Pyranones and derivatives	98.3
(2S)-2-[(3R,7R,8R,8aS)-3,4'-dihydroxy-4,4,7,8a-tetramethyl-6'-oxospiro[2,3,4a,5,6,7-hexahydro-1H-naphthalene-8,2'-3,8-dihydrofuro[2,3-e]isoindole]-7'-yl]pentanedioic acid	10.50507	516.2695	[M+H]+	516.2675	C28H37NO8	Glutamic acid and derivatives	98.2
Isosafrole	10.66522	185.0622	[M+H]+	185.0573	C10H10O2	Benzodioxoles	88.8
Hosenkoside M	10.83022	1111.591	[M+H]+	1111.59	C53H90O24	Triterpenoids	100
[6-acetyloxy-7-hydroxy-1-(3-methylbutanoyloxy)-7-(3-methylbutanoyloxymethyl)-4a,5,6,7a-tetrahydro-1H-cyclopenta[c]pyran-4-yl]methyl 3-methylbutanoate	10.83022	544.3082	[M+H]+	544.312	C27H42O10	Tetracarboxylic acids and derivatives	94
Ponatinib (AP24534)	11.07003	533.2271	[M+H]+	533.227	C29H27F3N6O	Benzanilides	100
isoabienol	11.15087	313.2502	[M+H]+	313.25	C20H34O	Diterpenoids	100
(2E,4E)-12-[(10E,12E)-13-carboxy-3-[(2E,4E)-13-carboxy-12,14-dihydroxy-3,5,7-trimethyltetradeca-2,4-dienoyl]oxy-2-(hydroxymethyl)-8,10,12-trimethyltrideca-10,12-dienoyl]oxy-13-(hydroxymethyl)-3,5,7-trimethyltetradeca-2,4-dienedioic acid	11.15087	1013.582	[M+H]+	1013.581	C54H86O16	3-(3-hydroxyalkanoyloxy)alkanoic acids	99.9
(2E,6E,11E)-18-(2,6-dioxopiperidin-4-yl)-9,13-dihydroxy-8-methoxy-10,12,14-trimethyl-15-oxooctadeca-2,6,11-trienoic acid	11.27018	546.3173	[M+H]+	546.3149	C27H41NO8	Lineolic acids and derivatives	97.8
[(2R,3S,4S,5R,6S)-3,4,5-trihydroxy-6-[(2S,3R,4S,5S,6R)-3,4,5-trihydroxy-6-	11.27018	528.3067	[M+H]+	528.302	C23H42O12	Saccharolipids	90.6

(hydroxymethyl)oxan-2-yl]oxyoxan-2-yl]methyl 9-methyldecanoate							
LPE 18:2	11.43085	478.2964	[M+H]+	478.2945	C23H44NO7P	Lipids	98.2
(1S,4R,5R,6S,8R,10R,13S,16S,18R)-4-(acetyloxy)-11-ethyl-16-hydroxy-6,18-dimethoxy-13-(methoxymethyl)-11-azahexacyclo[7.7.2.1?,?.0?,??.0?,?.0??,??]nonadecan-8-yl acetate	11.67083	522.3171	[M+H]+	522.3167	C28H43NO8		99.9
5-amino-2-(3,4'-dihydroxy-4,4,7,8a-tetramethyl-6'-oxospiro[2,3,4a,5,6,7-hexahydro-1H-naphthalene-8,2'-3,8-dihydrofuro[2,3-e]isoindole]-7'-yl)-5-oxopentanoic acid	12.0713	532.3024	[M+H]+	532.3017	C28H38N2O7	Glutamine and derivatives	99.8
[(2R)-2-[(E,2S,4R)-4,6-dimethyloct-6-en-2-yl]-6-oxo-2,3-dihydropyran-3-yl] (2E,4E,6S)-8-hydroxy-6-(hydroxymethyl)-4-methylocta-2,4-dienoate	12.0713	473.2292	[M+H]+	473.23	C25H38O6	Fatty alcohols	99.7
THIAMINE	12.31847	283.1472	[M+H]+	283.1461	[C12H17N4OS]+	Thiamines	99.4
12-(acetyloxy)-6-(furan-3-yl)-14-hydroxy-1,7,11,15,15-pentamethyl-5-oxo-3-oxapentacyclo[8.8.0.0?,?.0?,?.0??,??]octadecan-18-yl acetate	12.47945	546.3103	[M+H]+	546.3062	C30H40O8	Limonoids	91.7
CUDA* (internal standard)	12.47945	341.2822	[M+H]+	341.2775	C19H36N2O3		89.4
6-Hydroxycaproic acid	12.59928	133.0905	[M+H]+	133.0859	C6H12O3	Medium-chain hydroxy acids and derivatives	90
1-(9-hydroxy-2-isobutyl-10-(3-(pyridin-3-ylmethoxy)phenyl)pyrrolo[3',4':6,7]azepino[4,3,2-cd]indol-8(2H,7H,10H)-yl)ethanone	12.59928	507.2357	[M+H]+	507.24	C31H30N4O3	Benzazepines	80.5
1,6-dihydroxy-8-(hydroxymethyl)-4,12,12,15-	12.89068	550.3433	[M+H]+	550.3374	C30H44O8	Phorbol esters	86.5

tetramethyl-14-[(2-methylpropanoyl)oxy]-5-oxotetracyclo[8.5.0.0?,?.0??,??]pentadeca-3,8-dien-13-yl 2-ethylbutanoate							
Celastrol	13.13092	473.2682	[M+H]+	473.2662	C29H38O4	Triterpenoids	98.1
(2R)-8-[(2R,3S)-5,7-dihydroxy-2-(4-hydroxyphenyl)-4-oxo-2,3-dihydrochromen-3-yl]-5,7-dihydroxy-2-(4-hydroxyphenyl)-2,3-dihydrochromen-4-one	13.21142	581.2032	[M+H]+	581.1977	C30H22O10	Biflavonoids and polyflavonoids	89.4
2-[(6,7-dimethoxy-3,4-dihydroisoquinolin-1-yl)methyl]-3-ethyl-9,10-dimethoxy-2,3,4,6,7,11b-hexahydro-1H-benzo[a]quinolizine	13.41273	501.2614	[M+H]+	501.2629	C29H38N2O4	Emetine alkaloids	98.8
LPC 18:2	13.41273	520.3372	[M+H]+	520.3408	C26H50NO7P	Lipids	81.9
Polygalic Acid	14.631	489.3227	[M+H]+	489.32	C29H44O6	12-alpha-hydroxysteroids	96.4
9-stearolic acid	13.61463	279.263	[M-H]-	279.2634	C18H32O2	Long-chain fatty acids	99.9
Phosphatidylcholine lyso 19	12.76633	568.3994	[M-H]-	568.3984	C27H56NO7P	2-acyl-sn-glycero-3-phosphocholines	99.6

Supplementary Table S2. The components with a total score higher than 80 in the drug-containing serum of EAdSc.

Title	RT (min)	Precursor m/z	Adduct	Reference m/z	Formula	Ontology	Total score
2-(2-(2,5-dioxohexahydroimidazo[4,5-d]imidazol-1(2H)-yl)acetamido)acetic acid	1.33795	280.0717	[M+H]+	280.07	C8H11N5O5	N-acyl-alpha amino acids	98.6
anthothecol	5.247967	481.2296	[M+H]+	481.22	C28H32O7	Limonoids	80.9
4-PYRIDOXATE	6.891567	184.0596	[M+H]+	184.06	C8H9NO4	Pyridinecarboxylic acids	99.9
(1S,2R,4S,9R,10R,14S,15S,17S)-9-(furan-3-yl)-1-hydroxy-15-[(1R)-1-hydroxy-2-methoxy-2-oxoethyl]-10,14,16,16-tetramethyl-7,18-dioxo-3,8-dioxapentacyclo[12.3.1.0?,?.0?,??.0?,??]octadecan-17-yl	6.891567	592.279	[M+H]+	592.2752	C30H38O11	Limonoids	95

propanoate							
2-[(4-ethyl-8,8-dimethyl-2-oxo-9,10-dihydropyrano[2,3-h]chromen-5-yl)oxy]-N-(furan-2-ylmethyl)acetamide	6.971817	412.1805	[M+H]+	412.176	C23H25NO6		90.3
4-[[[2-[(8,8-dimethyl-2-oxo-4-propyl-9,10-dihydropyrano[2,3-h]chromen-5-yl)oxy]acetyl]amino]methyl]cyclohexane-1-carboxylic acid	7.51045	486.2485	[M+H]+	486.249	C27H35NO7		99.9
Tripterifordin	8.068583	319.2198	[M+H]+	319.22	C20H30O3	Diterpene lactones	100
Eicosanoids_12,13diHOME_C18H34O4	8.068583	337.2287	[M+H]+	337.235			82.2
Perindopril Erbumine (Aceon)	8.14925	391.2188	[M+H]+	391.22	C23H43N3O5	Dipeptides	99.3
ANTIMYCIN A (A1 shown)	8.155084	552.292	[M+H]+	552.2916	C27H38N2O9	Acylaminobenzoic acid and derivatives	99.9
NETILMICIN SULFATE	8.195084	574.2703	[M+H]+	574.2753	C21H43N5O11S	Aminocyclitol glycosides	90.9
Dihydrojasmonic Acid	8.240916	235.1305	[M+H]+	235.1305	C12H20O3	Jasmonic acids	100
(9Z,12E)-15,16-dihydroxyoctadeca-9,12-dienoic acid	8.240916	335.2125	[M+H]+	335.2157	C18H32O4		94.9
N-[(2S)-1-[(2-amino-2-oxoethyl)amino]-4-methyl-1-oxopentan-2-yl]-1-[1-(4-methylphenyl)sulfonylpiperidine-4-carbonyl]pyrrolidine-2-carboxamide	8.729733	550.2736	[M+H]+	550.27	C26H39N5O6S		94.8
Lobeline Hydrochloride	8.810217	355.2369	[M+H]+	355.238	C22H27NO2	Alkyl-phenylketones	99.4
METHYLPREDNISOLONE	8.810217	375.2241	[M+H]+	375.219	C22H30O5	21-hydroxysteroids	87.7
2-(7-hydroxy-6-methyloctyl)-2H-furan-5-one	8.85055	227.1615	[M+H]+	227.164	C13H22O3	Fatty alcohols	97
methyl (2E,8E)-9-[3a-hydroxy-6,7-dimethyl-1-(2-methylpropyl)-3-oxo-2,4,7,7a-tetrahydro-1H-isoindol-4-yl]-4,5-dihydroxy-8-methylnona-2,8-dienoate	8.942034	472.2711	[M+H]+	472.2721	C25H39NO6	Isoindolones	99.4
(4S,5Z,6S)-4-(2-methoxy-2-oxoethyl)-5-[2-[(E)-3-phenylprop-2-enoyl]oxyethylidene]-6-[(2S,3R,4S,5S,6R)-3,4,5-trihydroxy-6-(hydroxymethyl)oxan-2-yl]oxy-4H-pyran-3-carboxylic acid	9.1427	342.1837	[M+H]+	342.187	C16H23NO7	Pyrrolizines	94.6

2,7,7,11,15,17-hexamethyl-18-methylidene-5,13,16-trioxo-6,14-dioxatetracyclo[9.8.0.0?,?.0??,??]nonadec-3-en-10-yl acetate	9.3091	497.1905	[M+H]+	497.1936	C26H34O7	Naphthopyrans	95.2
3-[3-[3,4-dihydroxy-5-(6-oxo-3H-purin-9-yl)oxolan-2-yl]propanoyl]benzoic acid	9.646833	437.1621	[M+H]+	437.162	C19H18N4O7	5'-deoxyribonucleosides	100
3-(2,4-dihydroxyphenyl)-7-hydroxy-6,8-bis(3-methylbut-2-enyl)-2,3-dihydrochromen-4-one	10.2308	431.1868	[M+H]+	431.1851	C25H28O5	8-prenylated isoflavanones	98.6
TOBRAMYCIN	10.2308	490.2536	[M+H]+	490.2483	C18H37N5O9	4,6-disubstituted 2-deoxystreptamines	87
(1R,2R,4aS,6aS,6bR,9R,10R,11R,12aR)-1,10,11-trihydroxy-9-(hydroxymethyl)-1,2,6a,6b,9,12a-hexamethyl-2,3,4,5,6,6a,7,8,8a,10,11,12,13,14b-tetradecahydropicene-4a-carboxylic acid	10.27113	505.1948	[M+H]+	505.191	C30H48O6	Triterpenoids	93.1
methyl 8-hydroxy-4,5,7,10,14,14-hexamethyl-6,17-dioxo-16-oxapentacyclo[13.2.2.0?,??.0?,??.0?,?]nonadeca-3,7-diene-9-carboxylate	10.39062	481.2003	[M+H]+	481.1987	C26H34O6	Naphthopyrans	98.7
Ulipristal	10.51628	476.2796	[M+H]+	476.28	C30H37NO4	Steroid esters	99.9
methyl (4R,8aS)-1-hydroxy-2-(hydroxymethyl)-5,5,8a-trimethyl-4-[(2E,4E,6E)-octa-2,4,6-trienoyl]oxy-4a,6,7,8-tetrahydro-4H-naphthalene-1-carboxylate	10.51628	457.2004	[M+H]+	457.1987	C24H34O6	Fatty acid esters	98.4
Sibiromycin-494 hemiaminal	10.51628	494.2882	[M+H]+	494.286	C25H39N3O7	Aminoglycosides	97.7
[(1R,5R,9S,13S)-5,9,13-trimethyltetracyclo[11.2.1.0?,??.0?,?]hexadec-14-en-5-yl]methanol	10.51628	311.2368	[M+H]+	311.2345	C20H32O	Diterpenoids	97.3
(2S)-2-[(3R,7R,8R,8aS)-3,4'-dihydroxy-4,4,7,8a-tetramethyl-6'-oxospiro[2,3,4a,5,6,7-hexahydro-1H-naphthalene-8,2'-3,8-dihydrofuro[2,3-e]isoindole]-7'-yl]pentanedioic acid	10.51628	516.2716	[M+H]+	516.2675	C28H37NO8	Glutamic acid and derivatives	92.5
(2S,3R,4S,5S,6R)-2-[(2R,3R,4S,5S,6R)-4,5-dihydroxy-6-	10.63595	1061.585	[M+H]+	1061.59	C53H90O22	Triterpene saponins	88.2

(hydroxymethyl)-2-[[(3S,8R,10R,12R,14R,17S)-12-hydroxy-4,4,8,10,14-pentamethyl-17-[(2S)-6-methyl-2-[(2S,3R,4S,5S,6R)-3,4,5-trihydroxy-6-[[(2S,3R,4S,5S)-3,4,5-trihydroxyoxan-2-yl]oxymethyl]oxan-2-yl]oxyhept-5-en-2-yl]-2,3,5,6,7,9,11,12,13,15,16,17-dodecahydro-1H-cyclopenta[a]phenanthren-3-yl]oxy]oxan-3-yl]oxy-6-(hydroxymethyl)oxane-3,4,5-triol							
4-Methylabyssinone V	10.7956	445.197	[M+H]+	445.1985	C26H30O5	3'-prenylated flavanones	98.8
(2E,4E)-12-[(10E,12E)-13-carboxy-3-[(2E,4E)-13-carboxy-12,14-dihydroxy-3,5,7-trimethyltetradeca-2,4-dienoyl]oxy-2-(hydroxymethyl)-8,10,12-trimethyltrideca-10,12-dienoyl]oxy-13-(hydroxymethyl)-3,5,7-trimethyltetradeca-2,4-dienedioic acid	11.16142	991.6041	[M+H]+	991.599	C54H86O16	3-(3-hydroxyalkanoyloxy)alkanoic acids	96.7
(2E,6E,11E)-18-(2,6-dioxopiperidin-4-yl)-9,13-dihydroxy-8-methoxy-10,12,14-trimethyl-15-oxooctadeca-2,6,11-trienoic acid	11.28125	546.3163	[M+H]+	546.3149	C27H41NO8	Lineolic acids and derivatives	99.2
Aluminum dimerumic acid [M+Al-2H]	11.28125	509.2234	[M+H]+	509.22	C22H36N4O8	Cyclic carboximidic acids	94.7
(2Z,6E,10Z)-12-acetyloxy-10-(acetyloxymethyl)-6-methyl-2-(4-methylpent-3-enyl)dodeca-2,6,10-trienoic acid	11.4014	459.2161	[M+H]+	459.22	C24H36O6	Acyclic diterpenoids	92.5
isoabienol	11.44157	313.2502	[M+H]+	313.25	C20H34O	Diterpenoids	100
LPE 18:2	11.44157	478.2965	[M+H]+	478.2945	C23H44NO7P	Lipids	98.1
(E)-3-(4-methoxyphenyl)-1-[2,4,6-trimethoxy-3-(3-methylbut-2-enyl)phenyl]prop-2-en-1-one	11.48123	419.2229	[M+H]+	419.2248	C24H28O5	3-prenylated chalcones	98.2
(1S,4R,5R,6S,8R,10R,13S,16S,18R)-4-(acetyloxy)-11-ethyl-16-hydroxy-6,18-dimethoxy-13-(methoxymethyl)-11-azahexacyclo[7.7.2.1?,?.0?,??.0?,?.0??,??]nonadecan-8-yl acetate	11.68188	522.3189	[M+H]+	522.3167	C28H43NO8		97.8
[(2R)-2-[(E,2S,4R)-4,6-dimethyloct-6-en-2-yl]-6-oxo-2,3-dihydropyran-3-yl] (2E,4E,6S)-8-hydroxy-6-(hydroxymethyl)-4-	12.08187	473.2306	[M+H]+	473.23	C25H38O6	Fatty alcohols	99.8

methylocta-2,4-dienoate							
Isosafrole	12.48552	185.0617	[M+H]+	185.0573	C10H10O2	Benzodioxoles	90.6
Celastrol	13.12847	473.2654	[M+H]+	473.2662	C29H38O4	Triterpenoids	99.7
Salinomycin, Sodium	13.2493	790.5088	[M+H]+	790.5076	C42H69NaO11	Diterpene glycosides	99.7
2-[(6,7-dimethoxy-3,4-dihydroisoquinolin-1-yl)methyl]-3-ethyl-9,10-dimethoxy-2,3,4,6,7,11b-hexahydro-1H-benzo[a]quinolizine	13.45145	501.2617	[M+H]+	501.2629	C29H38N2O4	Emetine alkaloids	99.3
(3S,4S,6aR,6bS,8R,8aR,12aS,14bR)-8-hydroxy-4,6a,6b,11,11,14b-hexamethyl-3-[(2S,3R,4S,5R)-3,4,5-trihydroxyoxan-2-yl]oxy-1,2,3,4a,5,6,7,8,9,10,12,12a,14,14a-tetradecahydropicene-4,8a-dicarboxylic acid	13.61262	657.3256	[M+H]+	657.3252	C35H54O10	Triterpenoids	100
(4E)-4-[3-[4,5-dihydroxy-6-(hydroxymethyl)-3-[3,4,5-trihydroxy-6-(hydroxymethyl)oxan-2-yl]oxyoxan-2-yl]oxy-14-hydroxy-10,13-dimethyl-2,3,4,5,6,7,8,9,11,12,15,16-dodecahydro-1H-cyclopenta[a]phenanthren-17-ylidene]oxolan-2-one	13.61262	716.3944	[M+H]+	716.3851	C35H54O14	Steroidal glycosides	81.1
[1,3,12-triacetyloxy-17-(furan-3-yl)-4,4,8,10,13-pentamethyl-2,3,5,6,7,9,11,12,16,17-decahydro-1H-cyclopenta[a]phenanthren-7-yl] 2-hydroxy-3-methylpentanoate	14.05742	688.4067	[M+H]+	688.406	C38H54O10	Limonoids	99.9
(3S,10R,13R)-10,13-dimethyl-17-octyl-2,3,4,7,8,9,10,11,12,13,14,15,16,17-tetradecahydro-1H-cyclopenta[a]phenanthren-3-yl (4-nitrophenyl) carbonate	14.09792	552.3605	[M+H]+	552.36	C34H49NO5	Androstane steroids	99.9
Orlistat	14.09792	534.351	[M+H]+	534.355	C29H53NO5	Leucine and derivatives	93.3
1,2,6b,9,9,12a-hexamethyl-4a-[3,4,5-trihydroxy-6-(hydroxymethyl)oxan-2-yl]oxycarbonyl-10-(3,4,5-trihydroxy-6-methyloxan-2-yl)oxy-2,3,4,5,6,6a,7,8,8a,10,11,12,13,14b-tetradecahydro-1H-picene-6a-carboxylic acid	14.38025	812.4857	[M+H]+	812.4791	C42H66O14	Triterpene saponins	92
Phosphatidylcholine lyso 15	10.23435	512.3351	[M-H]-	512.3358	C23H48NO7P	2-acyl-sn-glycero-3-	99.8

						phosphocholines	
2-[[3,21-dihydroxy-20-[3,4,5-trihydroxy-6-(hydroxymethyl)oxan-2-yl]oxydocosan-2-yl]amino]acetic acid	11.99708	592.4056	[M-H]-	592.407	C30H59NO10	Fatty acyl glycosides of mono- and disaccharides	99.4
Phosphatidylcholine lyso 18	12.07642	554.3841	[M-H]-	554.3828	C26H54NO7P	2-acyl-sn-glycero-3-phosphocholines	99.3
Alpha-Hydroxydeoxycholic Acid	9.269567	391.3154	[M-H]-	391.3138	C24H40O4		98.7
Phosphatidylcholine lyso 19	12.52338	568.4003	[M-H]-	568.3984	C27H56NO7P	2-acyl-sn-glycero-3-phosphocholines	98.6
Phosphatidylcholine lyso 16	10.79082	526.3549	[M-H]-	526.3514	C24H50NO7P	2-acyl-sn-glycero-3-phosphocholines	94.9

Supplementary Table S3. The core targets of EAdSc components against UC.

Target	MCC	MNC	Degree	Closeness	Betweenness
MAPK1	4928268	81	81	321.3333	10680.09
MAPK3	4912640	76	76	303.25	5728.47
SRC	175698	72	72	288.6667	4575.447
HSP90AA1	56747	59	62	284.5	5362.387
STAT3	5096036	61	61	291.0833	3612.471
AKT1	100742	57	57	285.3333	3454.67
PIK3CA	106138	54	54	289.9167	2521.606
EGFR	189340	45	47	287.5	3996.058
ESR1	20393	46	47	300.9167	5484.061
MAPK14	196583	45	46	301.25	4538.055
RELA	23144	41	45	271	3775.671
LCK	1030074	43	43	283.1667	2170.212
FYN	187747	40	43	276.75	3173.732
JUN	15102	41	43	270.4167	2937.575
RXRA	38794	39	41	270.3333	2513.929
PTPN11	4868940	40	40	268.4167	1182.57
JAK2	4193704	39	39	289.25	1858.461
HDAC1	5810	37	37	266.9167	2360.494
SYK	60984	36	36	292.0833	2528.492
PTPN1	615	28	35	301.5	5034.39
JAK3	4917862	34	34	291.75	1790.442
JAK1	4975764	33	33	269.25	583.7481
CDK1	13400	33	33	282.5833	2287.953
NR3C1	6277	32	33	278.6667	1951.932
ITGB3	2921	32	33	282.4167	2120.814
VEGFA	12198	32	32	250.6667	598.0093
PLCG1	1602	28	32	254.9167	981.9469
AR	471	31	32	286.6667	2689.245
STAT1	4469762	31	31	260.0833	619.969
MAPK8	3900	31	31	272.25	1017.466
IL6	4074150	28	30	266.5	1896.759
ITGAV	3452	30	30	262.1667	1101.419
IL2	476691	27	28	252.8333	377.5634
PPARA	731	27	28	277.1667	1918.086
KDR	2435	24	27	284.5	2530.598
MMP9	424	25	27	288.75	3205.235
NCOA2	5918	24	26	232.6667	669.4811
CDK2	5176	26	26	272.25	1365.721
HIF1A	2755	21	26	261.1667	2050.82
RXRB	37826	25	25	253.25	464.2749
PDGFRB	36114	25	25	261.5	438.8528
IGF1R	3132	23	25	272	1477.229

RPS6KB1	1920	25	25	264.0833	826.2886
PRKCA	1852	21	25	264	1504.964
MMP2	286	25	25	293.25	2965.086
MDM2	172	25	25	283.8333	1878.122
CASP3	155	22	25	260	1946.483
F2	105	22	25	277	3199.986
CDK4	2806	24	24	264.1667	945.5588
MMP1	379	23	24	281.3333	2970.625
GSK3B	121	21	24	264.9167	1671.056
PTGS1	93	15	24	269.25	3873.759
RXRG	38424	23	23	244.9167	271.3108
PTPN6	32188	23	23	256.75	644.4618
CDC25A	5187	19	23	263	1939.384
CYP19A1	1627	20	23	272.6667	2556.21
TERT	860	21	23	268.9167	1152.573
NOS2	536	17	23	264.6667	2137.87
MTOR	534	23	23	270.75	846.6068
PDPK1	506	21	23	261.4167	979.9434
PTPN2	19712	22	22	254.3333	517.7418
ERBB2	3409	21	22	267.3333	717.6873
MET	2514	20	22	275	1220.201
AKR1C3	1636	20	22	247	1226.381
PPARG	291	21	22	271.1667	1554.282
HSD11B1	45	15	22	279.3333	2615.838
MAPK11	12206	21	21	254.8333	273.2556
CCNA2	10648	21	21	247.3333	355.691
PPP2CA	1820	21	21	246.25	357.6443
PIK3CB	1796	21	21	257.0833	308.914
CYP3A4	168	17	21	240	1370.98
CDC25B	2327	19	20	271.8333	1292.5
CASP8	385	19	20	256.0833	840.6316
PTGS2	105	15	20	258.25	1522.422
APP	84	16	20	252	1175.039
CDK5	2478	19	19	260.9167	668.0745
ABL1	292	19	19	250.75	287.8677
HLA-DRB1	179	16	19	249.1667	1372.23
ESR2	107	16	19	274.9167	1344.073
CYP1A2	39	14	19	251.5	1874.513
NLRP3	24	6	19	252.25	2298.316
PRKCD	19364	16	18	252	233.5665
CCNB1	11918	18	18	239.9167	298.0887
CCNB2	11720	18	18	260.1667	698.249
AURKA	5992	18	18	267.0833	688.7083
BCL2	838	18	18	269.75	472.6899

INSR	241	17	18	263.1667	911.0858
BCL2L1	214	18	18	261.0833	622.3677
CYP1A1	166	18	18	233.0833	644.6051
ALOX5	159	17	18	268.25	1953.284
CYP17A1	1612	17	17	250.25	865.2215
AKT2	344	17	17	254.9167	479.2865
NGFR	52	15	17	233.8333	599.2668
AURKB	5807	13	16	257.5833	823.2461
HDAC3	5222	16	16	250.6667	433.6543
BTK	1705	15	16	252.4167	301.8941
MAPK9	486	16	16	255.5	282.6936
MMP3	183	15	16	272.3333	890.3283
NTRK1	94	16	16	257.8333	424.778
CBFB	33	6	16	242.3333	696.6431
CHEK1	2308	15	15	245	320.1928
ITGB2	904	15	15	251.3333	574.2576
FABP1	214	15	15	246.0833	538.4452
HMGCR	73	12	15	246.1667	670.1531
TOP2A	6607	9	14	240.6667	625.0997
PGR	1014	12	14	262.5	250.1849
TYMS	879	11	14	249.25	935.1274
FLT1	171	11	14	269.0833	797.3721
PLA2G4A	138	14	14	256.9167	444.3136
MAPK10	116	12	14	269.3333	754.3041
DNM1	86	8	14	247.5	764.9706
SHBG	37	13	14	253.75	726.1953
VDR	15124	9	13	249.75	541.2508
HSD17B2	1581	12	13	246.4167	826.7974
ITGA4	1000	13	13	247.6667	406.4178
MAP3K14	152	10	13	247	574.5951
PLAU	76	11	13	240.6667	435.752
SCD	41	12	13	251.6667	595.9686
AKR1B10	32	7	13	259.3333	1082.586
AKR1B1	29	8	13	241.1667	963.0932
TLR4	22	9	13	243.5	546.4892
PARP1	21	5	13	260	1786.838
ITK	785	11	12	245.1667	310.2268
ALOX15	124	12	12	247.4167	559.7491
CDK5R1	42	7	12	238.0833	380.1643
EPHX2	37	5	12	258.3333	1787.932
RIPK2	33	11	12	241.8333	487.4901
FABP4	29	9	12	241	338.9617
HDAC6	27	9	12	251.8333	926.2771
HSPA5	23	7	12	246.5833	659.0944

STAT6	749	8	11	235.8333	234.8263
MCL1	159	10	11	249.5833	355.7744
ROCK2	140	11	11	250.8333	357.9001
NR1H4	92	9	11	244.75	480.2005
XIAP	49	10	11	234.1667	250.5494
ILK	38	11	11	246.75	241.6997
HDAC2	35	10	11	248.1667	554.54
RORC	34	7	11	246.4167	432.9858
TRPV1	31	8	11	250.75	748.3004
CASP1	26	9	11	252.1667	766.8123
ADAM17	24	9	11	263.0833	358.9791
MMP13	17	6	11	255.8333	640.5407
BACE1	15	6	11	266	924.445
CPT1A	172	8	10	240.25	320.5187
BRAF	55	9	10	248.6667	266.0747
CYP1B1	31	9	10	232.6667	368.9256
CSNK1D	28	8	10	237.8333	461.4695
SERPINE1	27	9	10	237.1667	243.8096
CNR1	25	7	10	246.9167	561.5133
PTGES	20	6	10	252.5833	611.1743
PTGER1	20	6	10	246.8333	520.2456
CCR5	20	5	10	238.8333	385.6187
F10	16	6	10	256.0833	759.9172
EDNRA	16	6	10	246.75	267.1606
FDFT1	49	8	9	240.25	356.3234
DUT	33	8	9	246.9167	312.5326
CYP51A1	29	8	9	233.5	389.0422
CFTR	20	9	9	238.5833	389.2798
KDM1A	17	8	9	235.5833	497.7021
